# *Ecdysone receptor* autonomously controls germ cell differentiation in the *Drosophila* ovary

**DOI:** 10.1242/dev.205460

**Published:** 2026-07-29

**Authors:** Lauren E. Jung, Alexandria I. Warren, Changhong Yin, Weihua Huang, Allison C. Simmons, Samantha I. McDonald, Lindsay A. Swain, Victoria E. Garrido, Daniel N. Phipps, BiClaireline Cesar, Danielle S. Finger, Zhipeng Sun, Todd G. Nystul, Elizabeth T. Ables

**Affiliations:** ^1^ Department of Biology, East Carolina University, Greenville, NC 27858, USA; ^2^ Department of Pathology and Laboratory Medicine, Brody School of Medicine, Greenville, NC 27834, USA; ^3^ National Key Laboratory of Green Pesticide, South China Agricultural University, 510642 Guangzhou, China; ^4^ Departments of Anatomy and OB/Gyn, University of California San Francisco, San Francisco, CA 94143, USA

**Keywords:** Germline stem cell, Oogenesis, Steroid hormone, Ecdysone

## Abstract

The steroid hormone ecdysone controls *Drosophila* ovarian germline stem cell (GSC) maintenance and germ cell differentiation. Prior studies demonstrated that ecdysone regulates germ cell differentiation non-autonomously via the nuclear receptor Ecdysone receptor (EcR) in ovarian somatic cells. Although EcR is also expressed in germ cells, potential direct roles for EcR in the germline independent of the soma have not been examined. Here, we demonstrate that EcR functions autonomously in GSCs and cystoblasts to control germline differentiation. While depletion of *EcR* from GSCs mildly reduces their maintenance, overexpression of *EcR* specifically in GSCs and cystoblasts impedes germ cell differentiation, phenotypically resembling loss of function of *bag of marbles* (*bam*) and bone morphogenetic protein signaling constitutive activation. We propose that EcR functions as a transcriptional repressor in GSCs and cystoblasts to suppress differentiation but becomes derepressed as the ecdysone titer increases and expression of the co-repressor Smrter decreases in dividing germ cell cysts, providing temporal control over germline differentiation. These data support the model that germ cells integrate signals from multiple cell sources that spatially and temporally control their differentiation in response to local and physiological cues.

## Teaser

**Summary:** The nuclear receptor EcR modulates stem cell maintenance and differentiation in ovarian germ cells.

## INTRODUCTION

Physiological signals provide spatiotemporal cues linking cellular differentiation with tissue demand. For example, stem cells in the skin, muscle, mammary gland, and hematopoietic system respond to hormone fluctuations during puberty and pregnancy (Chhabra and Booth, 2021; de Morree and Rando, 2023; Hsu and Fuchs, 2022; Nakada et al., 2014). As primary receptors for steroid hormones and other lipophilic signals, nuclear receptors are key molecular nodes coordinating cellular responses to physiological changes. Crosstalk between nuclear receptors influences cell plasticity and heterogeneity; moreover, dysregulation of nuclear receptor signaling contributes to many disease states (BharathwajChetty et al., 2024; Simandi et al., 2013; Weikum et al., 2018). Despite the highly conserved structure and function of nuclear receptors, the molecular mechanisms by which they control cell differentiation are poorly understood.

**Fig. 1.**
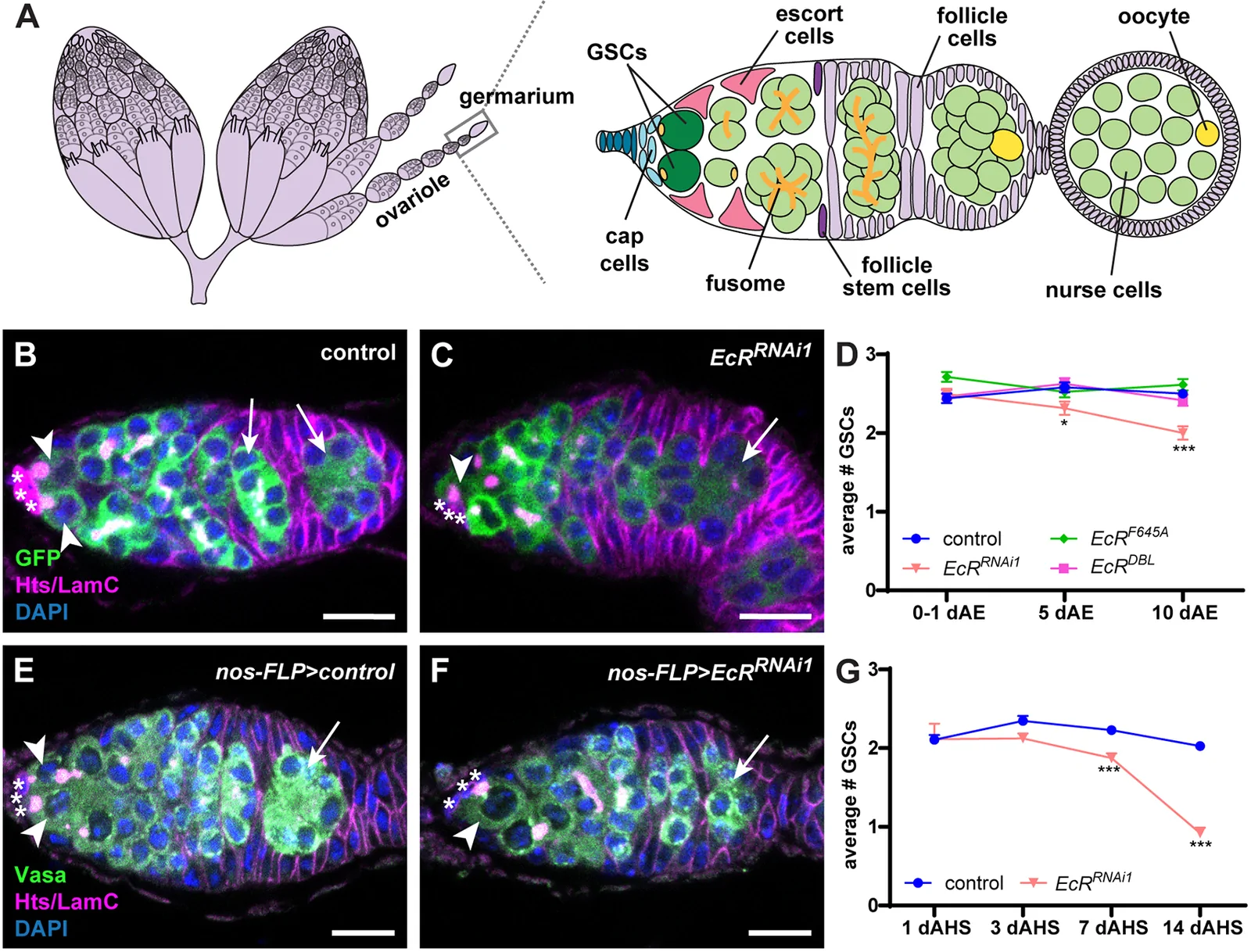
***EcR* is necessary for germline stem cell maintenance.** (A) Schematic of a *Drosophila* ovary. The germarium is the anterior-most region of each ovariole (boxed area enlarged at right). Germline stem cells **(**GSCs, dark green) divide to create cystoblasts and cysts (pale green), featuring the fusome (orange), a germ cell-specific organelle. The oocyte (yellow) differentiates from within the 16-cell cyst. Germ cells are surrounded by somatic cells: cap cells (light blue), escort cells (pink), follicle stem cells (purple) and follicle cells (pale purple). (B-D) Representative images (B,C) and quantification (D) of GSCs per germarium in *nos-Gal4::VP16>UASp-GFP::tubulin* control (B) and *nos-Gal4::VP16>UASp-GFP, EcR^RNAi1^* (C) germaria immunostained for GFP (green), Hts (magenta; fusomes and follicle cells), LamC (magenta; cap cells), and DAPI (blue; nuclei). dAE, days after eclosion; *n*>100 germaria per genotype per timepoint. (E-G) Representative images (E,F) and quantification (G) of germaria in *hsFLP;nos-STOP-Gal4* control (abbreviated *nos-FLP*) or *nos-FLP>EcR^RNAi1^* immunostained for Vasa (green), Hts and LamC (magenta), and DAPI (blue). dAHS, days after heat shock (DAHS); *n*>72 germaria per genotype per timepoint. Asterisks indicate cap cells, arrowheads indicate GSCs, and arrows indicate differentiated germ cells. Scale bars: 10 μm (B,C,E,F). Error bars show s.e.m. *P<0.01, ***P<0.0001, two-tailed, unpaired Student's *t*-test.

The *Drosophila* ovary provides an excellent model to study nuclear receptor-mediated control of cell differentiation (Hinnant et al., 2020; Spradling et al., 2022). Germline stem cells (GSCs) reside in the germarium, a niche at the anterior tip of the adult ovary, and supply progenitors for oocyte formation (Fig. 1A). GSCs divide asymmetrically to produce a cystoblast, which undergoes four rounds of mitotic division with incomplete cytokinesis to produce an interconnected 16-cell cyst. Within the cyst, one cell differentiates as the oocyte, while the remaining 15 become nurse cells that produce maternal mRNAs necessary for embryogenesis. Somatic cells surround GSCs and developing cysts and are essential for proper oocyte development and function.

GSC proliferation and maintenance depend on ecdysone, a steroid hormone well known to regulate developmental timing in insects (Ables and Drummond-Barbosa, 2010; Ables et al., 2016; Ameku and Niwa, 2016; König et al., 2011; Morris and Spradling, 2012; Shi et al., 2021). Ecdysone is synthesized from dietary cholesterol, and the ecdysone titer acts as a temporal cue to initiate developmental progression, pupariation, and metamorphosis (Morrow and Mirth, 2024). Ecdysone signals through the ecdysone receptor, a complex of two nuclear receptors [encoded by *EcR* (also known as *NR1H1*) and *usp* (also known as *NR2B4*)] that regulates gene expression (Morrow and Mirth, 2024; Uyehara and McKay, 2019). Cell type-specific responses are partly determined by differential expression and transcriptional activity of three EcR protein isoforms (EcR-A, EcR-B1, and EcR-B2) sharing conserved DNA- and ligand-binding domains (Schauer et al., 2011; Schubiger et al., 2003; Talbot et al., 1993). The ovary is the primary source of ecdysone in adult females, and ecdysone titers vary with age, reproductive status, and diet (Harshman et al., 1999; Hodgetts et al., 1977; Riddiford, 1993).

Global genetic loss-of-function studies indicate that ecdysone signaling promotes GSC self-renewal and cyst formation (Ables and Drummond-Barbosa, 2010; Morris and Spradling, 2012). While these processes are indirectly controlled by EcR activity in somatic cells (Ameku and Niwa, 2016; König and Shcherbata, 2015; König et al., 2011; Morris and Spradling, 2012; Shi et al., 2021), evidence suggests that EcR also functions autonomously in the early germline. First, germ cell-specific inactivation of the EcR obligate co-receptor encoded by *usp* blocked GSC self-renewal and cyst formation (Ables and Drummond-Barbosa, 2010). Second, several ecdysone-responsive genes, including other nuclear receptors, are expressed in germ cells (Ables et al., 2015; Beachum et al., 2021; Buszczak et al., 1999; McDonald et al., 2019; Zike et al., 2025). Finally, germ cell-specific inactivation of putative EcR target genes disrupted GSC self-renewal and germ cell differentiation (Ables et al., 2016). However, direct regulatory links between EcR and these transcriptional targets remain undefined.

Prior studies also suggest that EcR activity is coordinated with spatially restricted bone morphogenetic protein (BMP) signaling (Ables and Drummond-Barbosa, 2010; Drummond-Barbosa, 2019). BMP ligands are secreted from somatic cap cells and received by GSCs via transforming growth factor β (TGFβ)-class receptors, including the type I receptor encoded by *thickveins* (*tkv*). Signal reception induces phosphorylation and activation of the transcription factor encoded by *Mothers against decapentaplegic* (*Mad*), which represses transcription of *bag of marbles* (*bam*), encoding an essential differentiation factor. Asymmetric GSC division displaces the cystoblast farther from BMP ligands, enabling *bam* transcription. Mad also activates transcription of *Daughters against decapentaplegic* (*Dad*), which promotes degradation of active Tkv protein (Liu et al., 2023). Overexpression of constitutively active Tkv (*tkv^ACT^*) or knockdown of *bam* results in accumulation of undifferentiated germ cells (Casanueva and Ferguson, 2004; McKearin and Spradling, 1990; Xie and Spradling, 1998).

Here, we present evidence that EcR autonomously controls germline differentiation. Depletion of *EcR* mildly reduces GSC number, while overexpression of *EcR* in GSCs and cystoblasts delays germ cell differentiation, producing a mixed population of undifferentiated, proliferative germ cells that phenocopies loss of *bam* or constitutively active BMP signaling. We propose that in GSCs and cystoblasts, high levels of the co-repressor Smrter (Smr) enable EcR to function as a transcriptional repressor that suppresses germ cell differentiation. As cysts divide, Smr expression declines and ecdysone titers likely rise, derepressing EcR and permitting transcriptional activation of maternal genes. This activity, combined with the non-autonomous action of EcR in adjacent cap and escort cells, collectively modulates GSC retention in the niche and germ cell differentiation.

## RESULTS

### Depletion of *EcR* modestly reduces GSC maintenance

Because EcR and its obligate co-receptor Usp are ubiquitously expressed in the ovary (Buszczak et al., 1999; König et al., 2011; Morris and Spradling, 2012), we asked whether reducing *EcR* levels or EcR signaling in germ cells altered GSC retention in the niche. Using a germline-specific driver (*nos-Gal4::VP16*), we depleted *EcR* with germline-enhanced RNAi (*EcR^RNAi^*; Fig. S1) or inactivated EcR signaling by overexpressing new germline-permissive dominant-negative *EcR* transgenes carrying point mutations in the ligand-binding domain [*EcR-B1^F645A^*, *EcR-B1^W650A^*, or *EcR-B1^F645A+W650A^* (referred to as *EcR^DBL^*); Fig. S2]. Prior *in vitro* studies demonstrated that *EcR-B1^F645A^* and *EcR-B1^W650A^* fail to stimulate transcription and act as competitive inhibitors of the wild-type protein (Cherbas et al., 2003; Hu et al., 2003). Ubiquitous somatic expression of these transgenes caused larval lethality, confirming functionality (Fig. S2C), and germline expression of *EcR^RNAi^* or dominant-negative *EcR* reduced egg production equivalently (Fig. S1D). We counted GSCs, identified by their anteriorly localized fusomes, in germaria from age-matched females (Fig. 1B-D). Although expression of the dominant-negative *EcR* alleles did not significantly impact GSC number, *EcR* depletion caused a mild, progressive reduction in GSCs (Fig. 1D; Fig. S1E,F). These data indicate that reduced EcR levels, but not transcriptional activation, impair GSC maintenance. Since the effect on GSC number could reflect alterations in either GSC establishment or maintenance, we depleted *EcR* specifically at adulthood using inducible RNAi (Fig. 1E-G; Fig. S1F). Conditional depletion of *EcR* in germ cells post eclosion similarly reduced GSC number over time, indicating that EcR is necessary for GSC maintenance independent of its potential roles in germline establishment.

### Overexpression of *EcR* in GSCs and cystoblasts delays germ cell differentiation

**Fig. 2.**
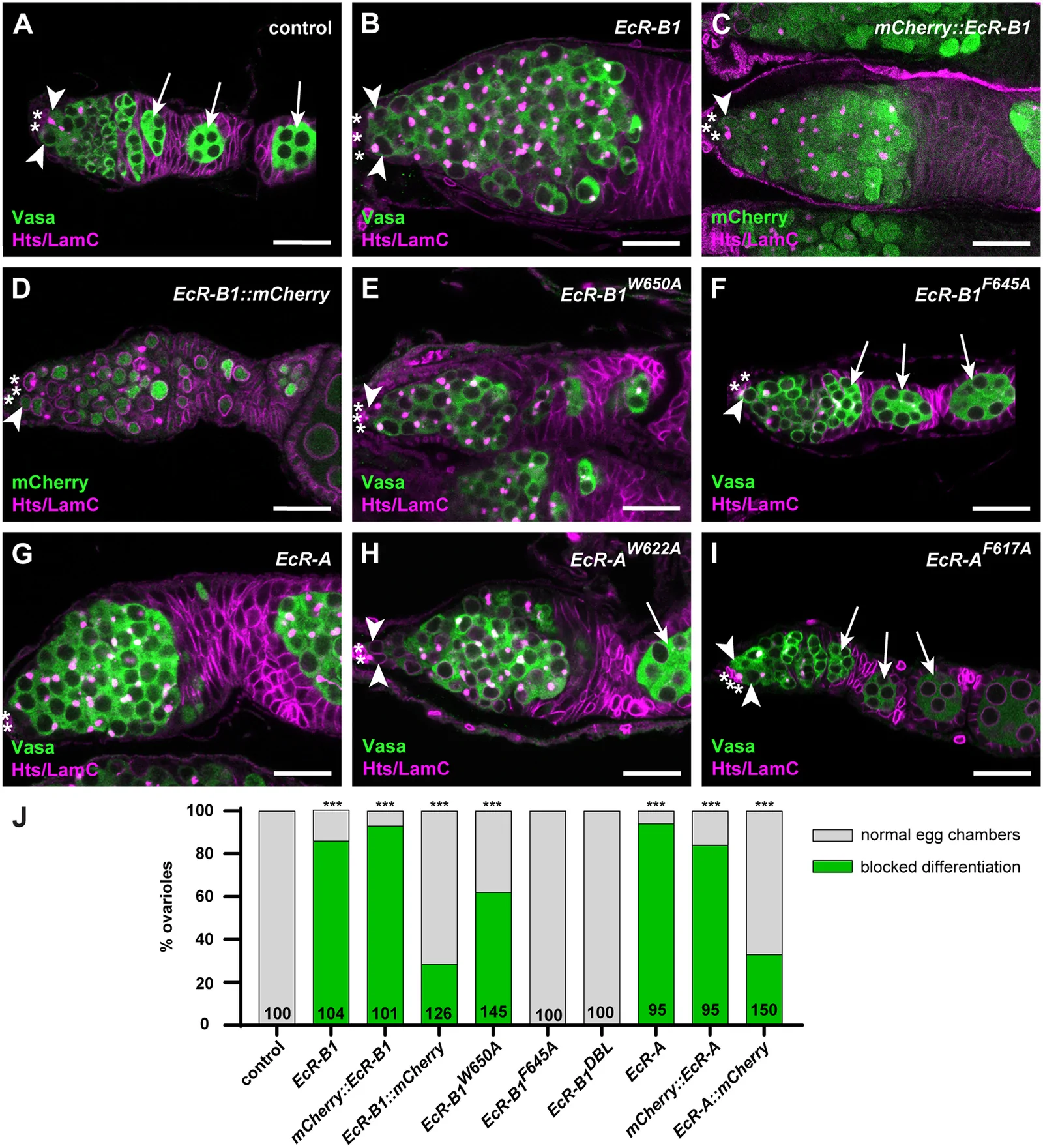
**Overexpression of *EcR* in germ cells impairs their differentiation.** (A-J) Representative images (A-I) and quantification (J) of germaria from *nos-Gal4::VP16* driving *UASp-GFP::tubulin* alone (A, control) or transgenic alleles of *EcR-B1* (B-F) or *EcR-A* (G-I) immunostained for Vasa (green, germ cells, A,B,E-I) or mCherry (green, transgene expression, C,D), Hts (magenta; fusomes and follicle cells), and LamC (magenta; cap cells and stalk cells). Scale bars: 20 μm. Asterisks indicate cap cells, arrowheads indicate GSCs, and arrows indicate differentiated germ cells. Bars in J represent the percentage of ovarioles with blocked germ cell differentiation (green); numbers in bars represent number of ovarioles scored from at least 15 flies per genotype, ∼5 days after eclosion. ***P<0.0001 compared to control; Fisher's exact test.

Since *EcR* is expressed at low levels in GSCs and cysts (Slaidina et al., 2021), we asked whether the levels of EcR must be kept low to permit germ cell differentiation. We overexpressed wild-type *EcR-B1* or a control *α-Tubulin* (*α-Tub::GFP*) transgene in germ cells and visualized germ cells with antibodies for the germline-enriched protein Vasa (Vas) (Fig. 2A,B). As expected, overexpression of *α-Tub::GFP* alone yielded fully developed ovaries containing two or three GSCs, mitotically dividing cysts, and developing egg chambers (Fig. 2A,J). In contrast, *EcR-B1* overexpression resulted in female sterility, with small ovaries lacking mature egg chambers and germaria filled with undifferentiated germ cells containing single round fusomes (Fig. 2B,J). Two additional independent transgenes in which EcR was terminally tagged with a fluorophore (Fig. 2C,D,J) produced similar phenotypes; however, expression of *EcR-B1::mCherry* blocked differentiation at lower penetrance, suggesting that the C-terminal fluorophore causes a conformational change in the protein, reducing function.

We then tested whether *EcR-B1* overexpression specifically in dividing cysts (exclusive of GSCs) could block germ cell differentiation. Using three independent transgenes that drive *Gal4* expression in dividing cysts (*3×bam-Gal4*), dividing cysts and 16-cell cysts (*aub-Gal4*), or post-mitotic cysts (*otu-Gal4::VP16*), we discovered that restricting overexpression of *EcR-B1* to the mitotic or post-mitotic cysts did not block their differentiation (Fig. S3). These results indicate that raising EcR protein titer specifically in GSCs and/or cystoblasts is sufficient to inhibit their differentiation.

Because EcR can both repress and activate transcription (Morrow and Mirth, 2024), we used the *EcR* dominant-negative alleles to further probe the mechanism of EcR action in early germ cells. We first quantified germline differentiation in females that overexpressed the germline-permissive dominant-negative forms of *EcR* (Fig. 2; Fig. S2). While expression of *EcR-B1^W650A^* specifically in germ cells (using *nos-Gal4::VP16*) partially blocked differentiation, expression of *EcR-B1^F645A^* or *EcR-B1^DBL^* did not block egg chamber formation (Fig. 2E,F,J). Expression of the *EcR-A* isoform or complementary versions of the dominant-negative *EcR-A* (*EcR-A^W622A^* and *EcR-A^F617A^*) recapitulated the *EcR-B1* phenotypes (Fig. 2G-J). Although both the *EcR-B1^F645A^* and *EcR-B1^W650A^* mutations are classified as dominant negative, prior studies indicate distinct mechanisms: *EcR-B1^F645A^* permits ligand binding but disrupts co-regulator interactions, whereas *EcR-B1^W650A^* blocks ligand binding (Cherbas et al., 2003; Hu et al., 2003). While we do not know the biochemical properties of the protein produced by the *EcR-B1^DBL^* allele, it alters the F645 and W650 amino acids simultaneously and genetically phenocopies *EcR-B1^F645A^*. These allele-specific effects support a model in which EcR primarily represses transcription in GSCs and cystoblasts and becomes derepressed and transcriptionally active in dividing cysts, likely in response to increased ecdysone levels.

### Germline-specific overexpression of *EcR* stalls germline differentiation, phenocopying constitutive activation of BMP signaling

**Fig. 3.**
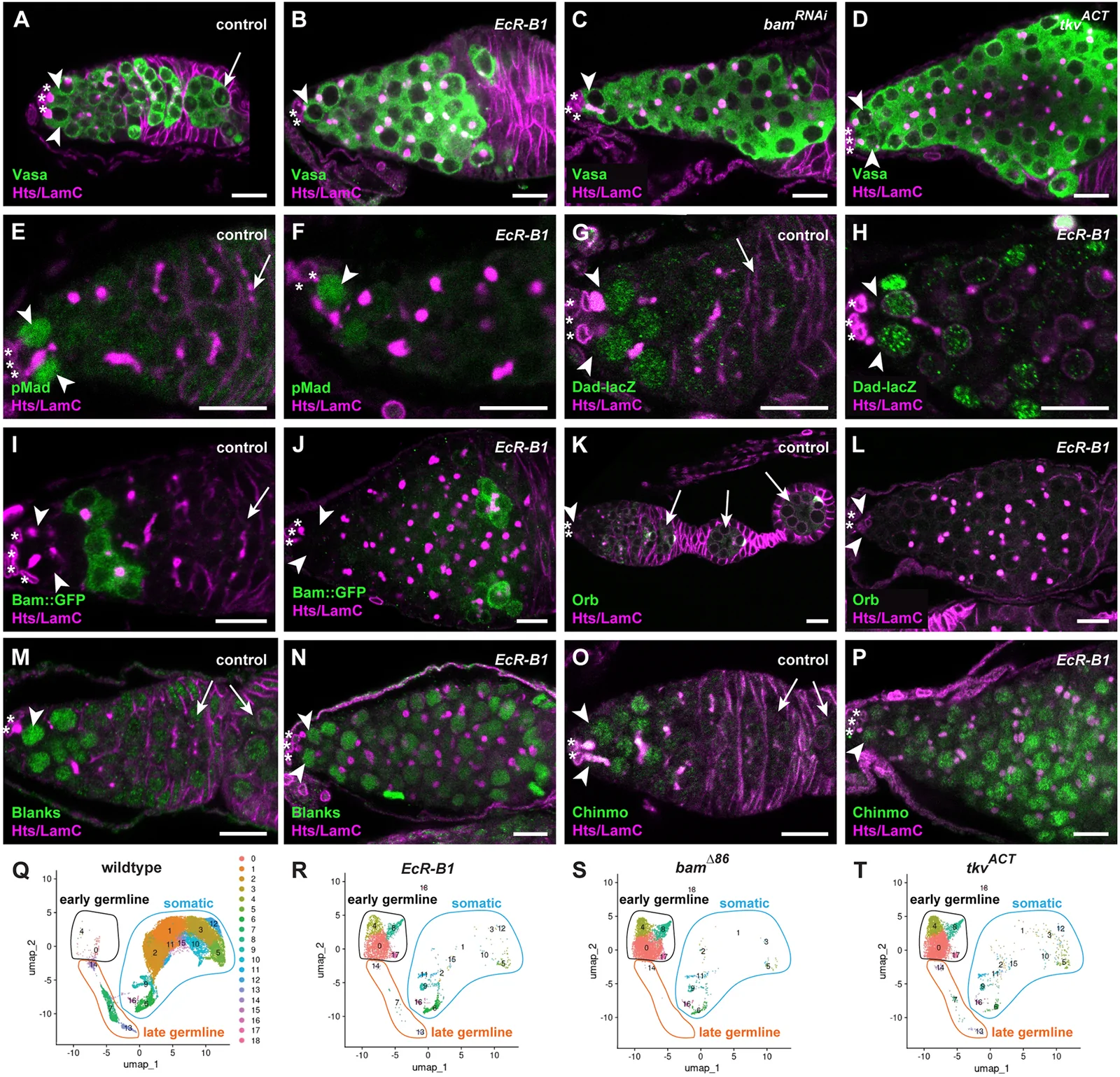
***EcR* overexpression phenocopies constitutive activation of BMP signaling.** (A-P) *nos-Gal4::VP16* driving *UASp-lacZ* control (A,E,G,I,K,M,O), *UASz-EcR-B1* (B,F,H,J,L,N,P), *bam^RNAi^* (C), or *UASp-tkv^ACT^* (D) germaria immunostained for Hts and LamC (magenta; fusomes, follicle cells, and cap cells), DAPI (blue, nuclei), and Vasa (green, germ cells, A-D), pMad (green, active BMP signaling, E,F), β-galactosidase (green, Dad reporter, G,H), GFP (green, Bam reporter, I,J), Orb (green, oocytes, K,L), Blanks (green, undifferentiated germ cells, M,N), or Chinmo (green, undifferentiated germ cells, O,P). Scale bars: 10 μm. Asterisks indicate cap cells, arrowheads indicate GSCs, and arrows indicate differentiated germ cells. Images represent at least ten germaria from at least five independent ovaries. (Q-T) Uniform manifold approximation and projection (UMAP) dimensionality reduction plots visualizing single-cell RNA sequencing analysis of all cell types from wild-type (Q, from Slaidina et al., 2021), *nos-Gal4::VP16>UASz-EcR-B1* (R), *bam^Δ86^* (S; from Sun et al., 2025 preprint), and *nos-Gal4::VP16>UASp-tkv^ACT^* (T) ovaries. Unique cell types (clusters) are numbered and displayed in differential colors, labeled for early germline (black outline), late germline (orange outline), or somatic cells (blue outline) based on differential gene expression (see Fig. S4 for specific markers).

Germline-specific overexpression of *EcR* yielded a phenotype resembling germline manipulations of BMP signaling (Casanueva and Ferguson, 2004; McKearin and Spradling, 1990; Xie and Spradling, 1998). Like *EcR*, overexpression of a constitutively active form of the BMP receptor Tkv (*tkv^ACT^*) or loss of function of the differentiation factor Bam (*bam^RNAi^* or *bam^Δ86^*) resulted in germaria full of single and doublet cells (Fig. 3A-D). Like wild-type ovaries, antibodies against phosphorylated Mad (pMad), a well-described readout of active BMP signaling, highlighted cells closest to cap cells in *EcR-B1* germaria (Fig. 3E,F). Similarly, cells lying closest to cap cells in *EcR-B1* ovaries expressed *Dad*, a transcriptional target of BMP signaling (Fig. 3G,H). While germ cells in *EcR-B1* ovaries rarely expressed *bam::GFP*, a reporter of *bam* expression (Fig. 3I,J), most did not express Orb, an RNA-binding protein enriched in oocytes in the final stages of mitotic division (Fig. 3K,L) (Hinnant et al., 2020). In contrast, *EcR-B1*-expressing germ cells co-expressed Blanks, a nuclear protein that is silenced in differentiating germ cells (Fig. 3M,N) (Kotb et al., 2024; Rust et al., 2020; Slaidina et al., 2021), and Chinmo, a transcription factor enriched in GSCs and undifferentiated germ cells (Fig. 3O,P) (Grmai et al., 2018).

To assess similarities in overall cellular composition between the genotypes, we used single-cell RNA sequencing (scRNA-seq) to compare ovaries overexpressing *EcR-B1* or *tkv^ACT^* with wild-type ovaries (Slaidina et al., 2021) and ovaries harboring a homozygous null mutation in *bam* (*bam^Δ86^*) (Sun et al., 2025 preprint). Germ cells were identified by enrichment for *nanos* (*nos*) and *vasa* and separated into undifferentiated and differentiated cells by expression of *Heterochromatin protein 6* (*HP6*) and *minidiscs* (*mnd*), respectively (Fig. S4) (Rust et al., 2020; Slaidina et al., 2021). Wild-type ovaries were primarily composed of somatic cells and contained few undifferentiated germ cells (Fig. 3Q). In contrast, *EcR-B1*, *bam^Δ86^*, and *tkv^ACT^* ovaries were enriched for undifferentiated germ cells and depleted of both differentiated germ cells and most somatic cells, consistent with the reduced size of the tissue (Fig. 3R-T). Taken together, these data indicate that overexpression of *EcR* in undifferentiated germ cells delays their differentiation prior to the mitotic-to-meiotic transition, phenocopying constitutively active *tkv* and *bam* loss of function.

### *EcR-B1* overexpression promotes *tkv* expression but does not block germ cell differentiation in a *tkv*- or *dpp*-dependent manner

**Fig. 4.**
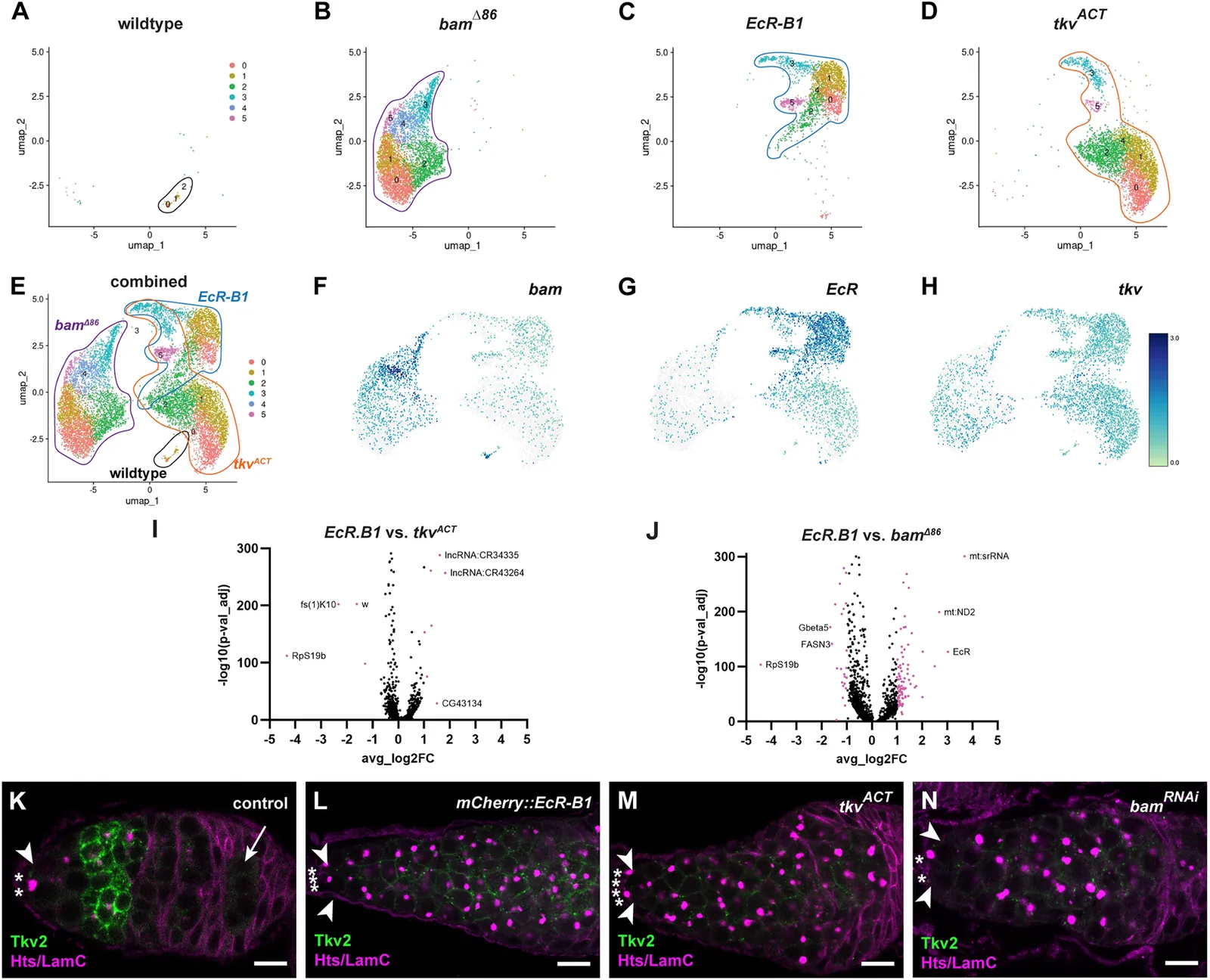
***EcR* overexpression promotes upregulation of *tkv* in undifferentiated germ cells.** (A-E) UMAP plots of undifferentiated germ cell sub-types from wild-type (A, from Slaidina et al., 2021), *bam^Δ86^* (B; from Sun et al., 2025 preprint), *nos-Gal4::VP16>UASz-EcR-B1* (C), and *nos-Gal4::VP16>UASp-tkv^ACT^* (D) ovaries. Six unique undifferentiated sub-types are displayed in differential colors (0, salmon; 1, gold; 2, green; 3, teal; 4, blue; 5, pink). A single UMAP of undifferentiated germ cell sub-types merged from all four genotypes is displayed in E, outlined for the individual genotypes (wild-type, black; *bam^Δ86^*, purple; *EcR-B1*, blue; *tkv^ACT^*, orange). (F-H) UMAP plots of undifferentiated germ cell subtypes overlayed with expression levels of *bam* (F), *EcR* (G), or *tkv* (H). Darker colors represent higher expression. (I,J) Volcano plots comparing differentially expressed genes in undifferentiated germ cell subtype 1 between *EcR-B1* and *tkv^ACT^* (I) and between *EcR-B1* and *bam^Δ86^* (J). (K-N) *nos-Gal4::VP16* driving *UASp-lacZ* control (K), *UASz-mCherry::EcR-B1* (L), *UASp-tkv^ACT^* (M), or *bam^RNAi^* (N) germaria immunostained with anti-Tkv2 antibodies (for Tkv, green) and Hts and LamC (magenta; fusomes, follicle cells, and cap cells). Scale bars: 10 μm. Asterisks indicate cap cells, arrowheads indicate GSCs, and arrows indicate differentiated germ cells. Images represent at least ten germaria from at least five independent ovaries.

To investigate transcriptional differences in germ cells overexpressing *EcR-B1* versus the other models, we used the scRNA-seq datasets to compare undifferentiated germ cells. We re-clustered the undifferentiated germ cells from wild-type, *bam^Δ86^*, *EcR-B1*, and *tkv^ACT^* germaria (combined, independent of the other cell types), resulting in six subtypes (Fig. 4A-E). Subtypes 0-3 included cells in wild-type ovaries, while subtypes 4 and 5 were unique, either to *bam^Δ86^* alone (subtype 4; Fig. 4B) or to all three genetic models (subtype 5; Fig. 4B-D). Although undifferentiated cells across the four genotypes had transcriptional profiles similar enough to cluster into subtypes, cells in most clusters shared higher similarity to cells of the same genotype; that is, undifferentiated cells in subtypes 0 and 1 clustered in four independent groups, based on the genotype of the cells (Fig. 4E). This is likely due to the differences in mRNA expression resulting from the genetic model itself. For example, *bam* mRNA was enriched across all clusters in *bam^Δ86^* cells (Fig. 4F), as the *bam^Δ86^* mutation blocks protein production but not mRNA production (McKearin and Spradling, 1990). Similarly, *EcR* mRNA was specifically enriched in *EcR-B1*-expressing cells (Fig. 4G).

Intriguingly, cells overexpressing *EcR-B1* or *tkv^ACT^* were highly similar, clustering together in overlapping profiles (Fig. 4C,D). This suggested that overexpression of *EcR-B1* or *tkv^ACT^* resulted in similar gene regulation in undifferentiated cells. While we expected that expression of *tkv* would be highest in *tkv^ACT^*-expressing cells, we found that *tkv* expression was upregulated in response to both *EcR-B1* and *tkv^ACT^* overexpression (Fig. 4H). Comparison of gene expression in individual subtypes revealed similar results. For example, few genes were differentially expressed in cells in subtype 1 between *EcR-B1* and *tkv^ACT^* germaria (Fig. 4I), while more genes were differentially expressed between *EcR-B1* and *bam^Δ86^* germaria (Fig. 4J).

To validate upregulation of *tkv* in *EcR-B1-*overexpressing cells *in situ*, we visualized Tkv protein levels in fixed tissue (Fig. 4K-N) (Peterson et al., 2022). Although wild-type GSCs express Tkv, activated Tkv is degraded (Liu et al., 2023). This results in relatively low levels of Tkv protein until the eight-cell cyst stage, where it localized at germ cell plasma membranes (Fig. 4K). Accordingly, *tkv^ACT^* and *bam^RNAi^* germaria displayed low levels of Tkv protein in undifferentiated germ cells, similar to wild-type GSCs (Fig. 4M,N). In contrast, Tkv protein was readily observed in *EcR-B1*-expressing germ cells away from the niche (Fig. 4L). Intriguingly, however, loss of *dpp* or *tkv* did not rescue *EcR*-induced differentiation defects, nor did depletion of *EcR* rescue a *tkv^ACT^*-mediated block to differentiation (Fig. S5). These data suggest that the EcR-mediated differentiation block is not dependent on BMP signaling.

### Undifferentiated germ cells from *EcR-B1*, *tkv^ACT^*, and *bam^Δ86^* ovaries are a mixed population of mitotic cells

While the undifferentiated germ cell subtypes 0 and 1 diverged based on genetic background, cells in subtypes 2 and 3 clustered together independent of genotype, suggesting that overexpression of *EcR-B1*, constitutive activation of *tkv*, or loss of *bam* yield cells that converge at a similar cell type (Fig. 4E). Consistent with this interpretation, computational temporal lineage analyses of the undifferentiated cell clusters based on pseudotemporal ordering or RNA kinetics [Monocle3 (Cao et al., 2019) and scVelo (Bergen et al., 2020), respectively] predict that subtypes 0 and 1 are most likely to be progenitors that differentiate into subtypes 2 and 3 (Fig. S6).

**Fig. 5.**
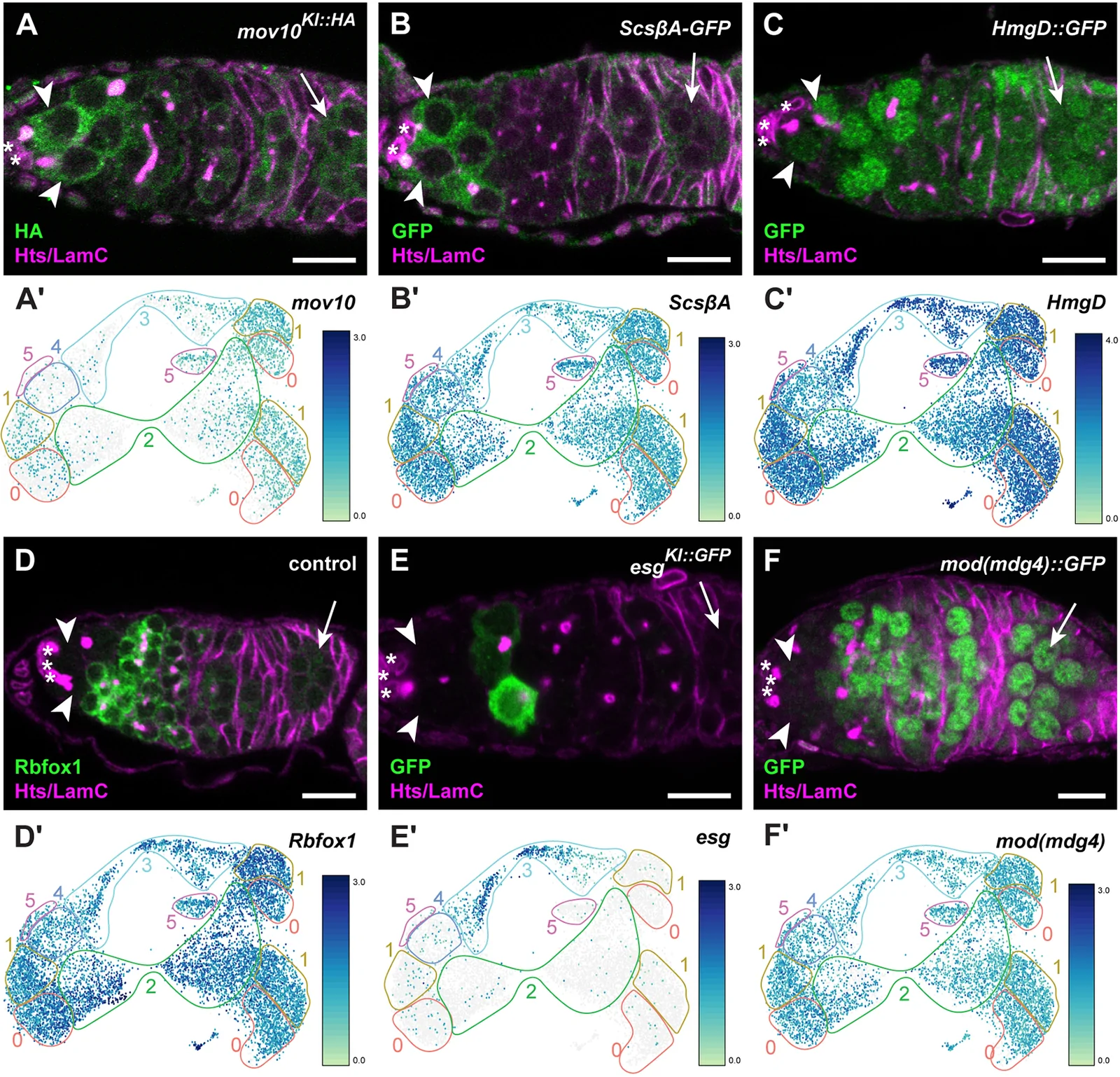
**Undifferentiated germ cells from *EcR-B1*, *tkv^ACT^*, and *bam^Δ86^* ovaries are a mixed population composed of GSCs, pre-cystoblasts, cystoblasts, and early dividing cysts.** (A-Fʹ) Representative images (A-F) and expression levels (A′-F′) in undifferentiated germ cells from all four genotypes in the merged UMAP (see Fig. 4E), visualizing *Mov10* (A,A′), ScsβA (B,B′), HmgD (C,C′), Rbfox1 (D,D′), *esg* (E,E′), and mod(mdg4) (F,F′). Germaria were immunostained for Hts and LamC (magenta; fusomes, follicle cells, and cap cells) and HA (A), GFP (B,C,E,F), or Rbfox1 (D) (green). Scale bars: 10 μm. Asterisks indicate cap cells, arrowheads indicate GSCs, and arrows indicate differentiated germ cells. Images represent at least ten germaria from at least five independent ovaries.

Cells in subtype 1 are enriched with genes known to be expressed in GSCs [*CG2887*, *CG9705*, *CG32814* (*eggplant* or *eggpl*), and *CG6628*; Rust et al., 2020; Slaidina et al., 2021; Sun et al., 2023, 2025] and to regulate GSC maintenance, such as the cell cycle factors encoded by *CycB* and *CycE* (Ables and Drummond-Barbosa, 2013; Wang and Lin, 2005) and the asymmetrically distributed small nucleolar ribonucleoprotein component encoded by *wicked* (*wcd*) (Fichelson et al., 2009). Gene Ontology analysis of subtype 1 indicates enrichment of genes associated with ‘protein localization’ and ‘RNA splicing’, consistent with GSCs. Furthermore, cell cycle analysis of the undifferentiated subtypes demonstrates that while all undifferentiated cells are proliferative, cells in subtype 1 are less likely to be found in the G1 phase of the cell cycle (Fig. S7), consistent with the short G1 length displayed by GSCs (Ables and Drummond-Barbosa, 2013; Hinnant et al., 2017). In contrast, subtype 0 does not express high levels of cell cycle-related genes and instead is associated with the ontologies ‘ATP synthesis’, ‘protein folding’, and ‘ribosome biogenesis’, associated with GSC differentiation (Sanchez et al., 2016; Teixeira et al., 2015). To corroborate the transcriptomics data, we visualized expression of three genes enriched in subtypes 0 and 1 (Fig. 5A-C′). The RNA helicase encoded by *Mov10* is enriched in subtype 1, consistent with endogenous expression in GSCs and pre-cystoblasts (Fig. 5A,A′) (Takemura et al., 2021). Similarly, levels of the metabolic enzyme encoded by *ScsβA* and the chromosome-associated protein encoded by *HmgD* were similar across the undifferentiated subtypes, including subtypes 0 and 1. While a fluorescent reporter of *HmgD* was expressed throughout the germline (with lower levels in GSCs in G2 of the cell cycle), an *ScsβA* reporter was enriched in GSCs and cystoblasts, consistent with additional post-transcriptional regulation (Fig. 5B-C′) (Buszczak et al., 2007). We conclude that subtypes 0 and 1 resemble endogenous GSCs or pre-cystoblasts.

Germ cells in subtypes 2 and 3 were enriched for genes expressed in dividing cystoblasts or cysts, including the translation regulator *Rbfox1* (Fig. 5D,D′) (Carreira-Rosario et al., 2016), and associated with the Gene Ontology terms ‘cytoplasmic translation’, ‘structural constituent of ribosome’, ‘transcription factor binding’, and ‘protein kinase activity’, which are hallmarks of the transition from stem cell to differentiated germ cell (Breznak et al., 2023). Surprisingly, gene expression was largely non-overlapping between subtypes 2 and 3. We identified examples of genes uniquely expressed in subtype 3, including the transcription factor encoded by *esg*, which is transiently expressed in cystoblasts (Fig. 5E,E′), despite its association with stem cell maintenance in other tissues (He et al., 2018). Many genes enriched in subtypes 2 and 3 are also highly expressed in differentiated germ cells. For example, expression of the chromatin regulator encoded by *mod(mdg4)* was equivalent across undifferentiated subtypes at the mRNA level; however, protein expression was suppressed in GSCs and enriched in differentiated cells (Fig. 5F,F′). Similarly, genes typically associated with differentiated germ cells, such as *Nlg2*, *mnd*, and *slif*, were expressed in subtypes 2 and 3 (Rust et al., 2020; Slaidina et al., 2021). Consistent with prior studies, these results suggest that some maternal genes are transcriptionally activated during the first cyst division, either to regulate cyst growth or to be deposited into the early embryo (Pang et al., 2023; Samuels et al., 2024).

To further assess the differentiation state of *EcR-B1* germ cells, we visualized the GSC- and cystoblast-enriched Mov10::HA and cyst-enriched Rbfox1 in *EcR-B1* germaria. As with Blanks and Chinmo (Fig. 3), Mov10::HA expression expanded past the stem cell niche in *EcR-B1* germaria (Fig. S8A). Conversely, cytoplasmic Rbfox1 protein was largely absent from nearly all single and doublet germ cells but could be easily visualized in the few cells that progressed into four-cell cysts (Fig. S8B). We conclude that *bam* loss of function, BMP constitutive activation, and *EcR* overexpression result in discrete populations of GSC-like cells (closest to cap cells), pre-cystoblast-like cells (anterior germaria), and early dividing cystocytes (posterior germaria) caught between the stem cell and maternal transcriptional programs.

Although germ cells in *bam* loss-of-function and constitutively active BMP genetic backgrounds have been used extensively as models for GSC-like cells, our comparative analyses raised two possibilities. First, all three genetic models could stall differentiation, rather than block it completely, resulting in a mixed population of cells at non-equivalent differentiation states. Alternatively, the genetic perturbations could result in a population of cells stuck in a differentiation transition state, expressing markers of both stem cells and differentiated cells. To reconcile these possibilities, we again used Monocle3 pseudotime computational analysis to track the lineage of cells in each genotype (Fig. S6D-F). While the trajectory of *EcR-B1* and *tkv^ACT^* germ cells ended in subtype 3, no endpoint could be identified in *bam^Δ86^* mutant germ cells. Instead, *bam^Δ86^* mutant germ cells exhibited a circuitous differentiation path, reverting to subtypes 0 and 1. These analyses suggest that *EcR-B1* and *tkv^ACT^* overexpression stall differentiation, while loss of *bam* pushes cells towards pre-cystoblast or cystoblast fates.

### A subset of canonical ecdysone-responsive genes is endogenously expressed in undifferentiated germ cells, suggesting a cell type-specific hormone response

**Fig. 6.**
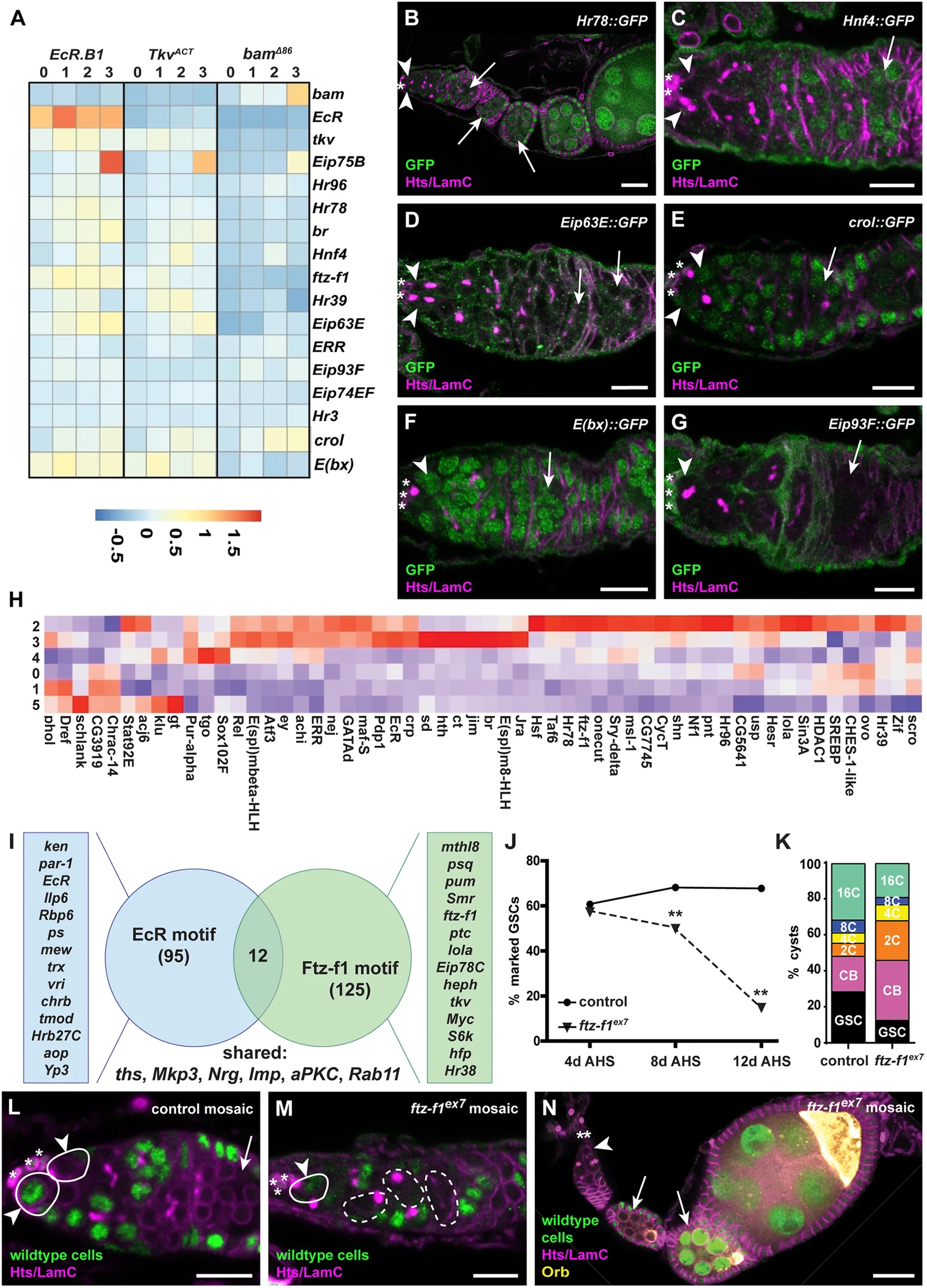
**EcR elicits a cell type-specific transcriptional response in germ cells.** (A) Heat map comparing expression of ecdysone-responsive genes in *nos-Gal4::VP16>UASz-EcR-B1*, *nos-Gal4::VP16>UASp-tkv^ACT^*, and *bam^Δ86^* undifferentiated germ cells (subtypes 0-3). (B-G) Representative germaria from GFP-tagged reporter lines for *Hr78* (B), *Hnf4* (C), *Eip63E* (D), *crol* (E), *E(bx)* (F), and *Eip93F* (G) immunostained for Hts and LamC (magenta; fusomes, follicle cells, and cap cells) and GFP (green, gene reporters). Images represent at least ten germaria from at least five independent ovaries. (H) Heat map showing transcription factor regulon enrichment in *bam^Δ86^* undifferentiated germ cells (all subtypes; see Table S4). (I) Comparison of putative EcR (blue) and Ftz-f1 (green) target genes in *bam^Δ86^* undifferentiated germ cells. Numbers represent the total number of differentially expressed genes containing a transcription factor-binding motif. (J-N) Lineage tracing using *flippase*-inducible mosaic analysis compares the fate of control (*FRT79D*) and *ftz-f1^ex7^* null mutant germ cells. GSC self-renewal (J) was measured in germline mosaic germaria from control (circles) and *ftz-f1^ex7^* mutants (triangles) as the percentage of total germaria with a GFP-negative GSC at 4, 8, and 12 days after clone induction (*n*>50 germaria per timepoint; **P<0.001, χ^2^ test). AHS, after heat shock. Cyst distribution (K) compared the number of marked GSCs, cystoblasts (CB), or cysts (2C, 4C, 8C, and 16C) in mock mosaic control (left) or *ftz-f1^ex7^* mutant (right) germaria. Panels L-N show representative images from mock mosaic controls (L; where some cells are lineage labeled by the absence of GFP but all cells are wild type) and *ftz-f1^ex7^* mutant ovarioles (M,N; where GFP-negative cells are homozygous null for *ftz-f1*) immunostained for GFP (green, wild-type cells), Hts and LamC (magenta), and Orb (yellow; N only). White outlines denote wild-type cells; dashed outlines denote *ftz-f1^ex7^* mutant cells. Images represent at least ten germaria from at least five independent ovaries. Scale bars: 10 μm (C-G,L,M); 20 μm (B,N). Asterisks indicate cap cells, arrowheads indicate GSCs, and arrows indicate differentiated germ cells.

EcR promotes cell type-specific transcriptional responses in target cells by repression and activation of a canonical transcriptional cascade of other nuclear receptors, chromatin-binding proteins, and transcription factors (Morrow and Mirth, 2024; Uyehara et al., 2022, 2019). To assess the degree to which the canonical EcR response is conserved in undifferentiated germ cells, we queried whether canonical ecdysone-responsive genes were differentially expressed in *EcR-B1*, *tkv^ACT^*, or *bam^Δ86^* cells (Fig. 6). We identified a subset of nuclear receptors and ecdysone-inducible genes upregulated in *EcR-B1* and *tkv^ACT^* cells (Fig. 6A). While prior studies demonstrated that *ftz-f1* is endogenously expressed in GSCs and dividing cysts and *Eip75B* is upregulated in 16-cell cysts, consistent with its enrichment in subtype 3 (Beachum et al., 2021; Buszczak et al., 1999), we confirmed that *Hr78*, *Hnf4*, *Eip63E*, *E(bx)*, and *crol* are also endogenously expressed in germ cells (Fig. 6B-F). Among these, *Eip75B* and *ftz-f1* are strong candidates as EcR target genes. *EcR-B1*-expressing germ cells were enriched for *ftz-f1* and *Eip75B* expression, and *Eip75B* expression increased with differentiation in all three genetic backgrounds (Fig. 6A). Depletion of *Eip75B* by germline-enhanced RNAi increased GSC number, while overexpression of *Eip75B* reduced GSC number (Fig. S9), supporting a role as a repressor of GSC self-renewal (Ables and Drummond-Barbosa, 2010).

Other known targets of EcR signaling, such as *ERR*, *Eip93F*, *Eip74EF*, and *Hr3*, were not differentially expressed between genotypes, suggesting that their regulation is independent of EcR. Indeed, *Eip93F*, which is essential for ecdysone-dependent transcription in other cell types (Uyehara et al., 2017), is expressed exclusively in ovarian somatic cells (Fig. 6G) and thus unlikely to mediate ecdysone-responsive transcription autonomously in germ cells. Notably, ecdysone biosynthesis gene expression was also absent from undifferentiated germ cells and was instead enriched in somatic cells of the germarium (Fig. S4L), supporting the model that escort cells provide a local source of ecdysone ligands (Shi et al., 2021).

Since *bam^Δ86^* cells appear to block, rather than delay, differentiation at the cystoblast stage, we used this dataset to further identify gene regulatory networks active at this transition independently of our EcR genetic manipulation. Using Single Cell rEgulatory Network Interference and Clustering (SCENIC) analysis (Aibar et al., 2017) to predict potential gene regulatory networks active specifically in *bam^Δ86^* cells, we noted that cells in subtypes 2 and 3 were enriched in transcripts regulated by EcR, the obligate co-receptor Usp, or EcR-dependent transcription factors (Hr39, Ftz-f1, Hr78, Hr96, and Br; Fig. 6H). As one example, we used DNA-binding motif enrichment analysis to ask which genes regulated by EcR could also be regulated by Ftz-f1 (Fig. 6I). Among 220 total enriched genes, only 12 contained both EcR and Ftz-f1 consensus binding sites, suggesting cascading regulation rather than co-regulation. Importantly, putative Ftz-f1 target genes included the transcriptional co-repressor encoded by *Smr*, which is enriched in undifferentiated germ cells (Fig. S10), and the nuclear receptor *Eip78C*, which is essential for cyst growth, packaging, and survival (Ables et al., 2015; Shi et al., 2021).

Taken together, gene regulatory network analyses suggest that EcR controls a transcription factor cascade during the earliest steps of germ cell differentiation. One prediction of this model is that an EcR-regulated transcription factor could impact both GSC self-renewal and cyst differentiation. To test the model, we used clonal analysis to trace the lineage of *ftz-f1* null mutant germ cells (Fig. 6J-N). We found that *ftz-f1* null mutant GSCs did not stay associated with the GSC niche over time, resulting in germaria lacking *ftz-f1* mutant GSCs but filled with single or doublet mutant germ cells (Fig. 6J,M). While *ftz-f1* mutant germ cells could differentiate into 16-cell cysts (Fig. 6N), their mitotic progression was greatly slowed, resulting in more mutant undifferentiated cells per germarium than controls (Fig. 6K). We conclude that *ftz-f1* is essential for GSC self-renewal and germ cell differentiation and may regulate gene expression downstream of EcR.

### EcR and BMP signaling co-regulate a shared transcriptional program

**Fig. 7.**
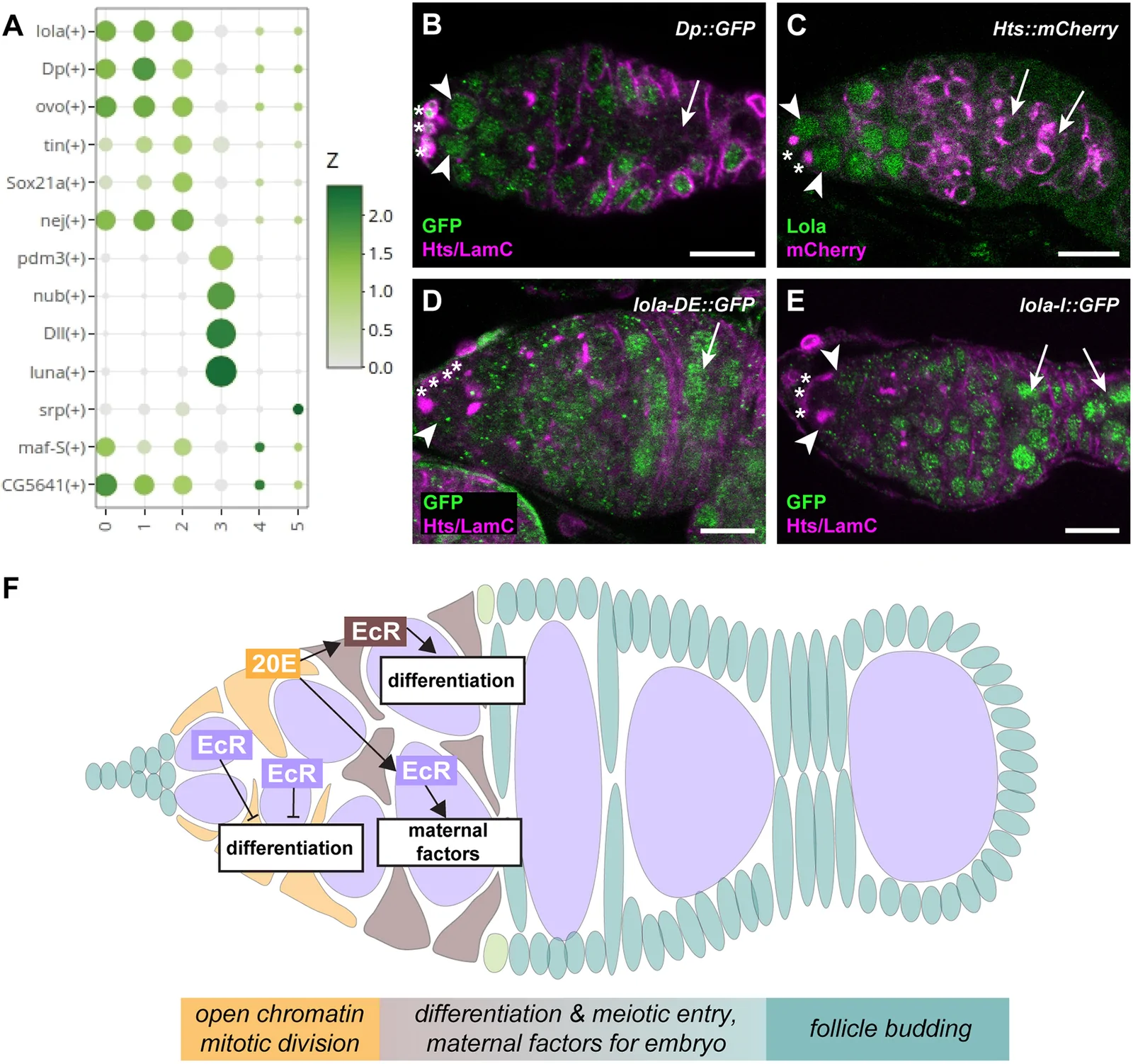
**EcR and BMP signaling co-regulate a basal transcription program controlling cell cycle progression and differentiation.** (A) Dot plot showing transcription factor regulon enrichment (regulon specificity score) in undifferentiated germ cells merged from all four genotypes (see Table S4). (B-E) Representative images of germaria from Dp (B) or Lola reporters (isoform *lola-DE*, D; isoform *lola-I*, E) immunostained for Hts and LamC (magenta; fusomes, follicle cells, and cap cells) and GFP (green, gene reporters), or wild-type germarium immunostained for Hts and LamC (magenta) and Lola (magenta). Scale bars: 10 μm. Asterisks indicate cap cells, arrowheads indicate GSCs, and arrows indicate differentiated germ cells. (F) Proposed model for EcR regulation of GSC maintenance and germ cell differentiation. EcR likely functions as a transcriptional repressor in GSCs and cystoblasts, where it acts to suppress differentiation. In differentiating cysts, EcR becomes derepressed and functions as a transcriptional activator as ecdysone titer (produced by neighboring escort cells) increases. This activity, combined with the non-autonomous action of EcR in adjacent cap and escort cells to promote germ cell differentiation, modulates GSC maintenance and germ cell differentiation.

The overlap in transcriptional repertoire between *EcR-B1*, *tkv^ACT^*, and *bam^Δ86^* undifferentiated germ cells suggested that EcR and BMP signaling regulate expression of similar genes in GSCs and dividing cysts. To identify potential regulatory programs downstream of EcR and BMP signaling, we combined all three scRNA-seq undifferentiated germ cell datasets and asked whether differentially expressed genes were enriched for common DNA-binding factor motifs using SCENIC analysis (Fig. 7A). Importantly, the predicted transcription factors controlling subtypes 0 and 1 included known regulators of self-renewal in the germline [i.e. Dp, Ovo, Nejire (Nej), and Maf-S] (Benner et al., 2024; Boija et al., 2017; Dynlacht et al., 1994; Kirilly et al., 2011; Pang et al., 2023; Tan et al., 2018). Among these, Dp binds the transcription factor E2F (also known as E2f1) to promote cell cycle progression and is expressed primarily in dividing germ cells (Fig. 7B), Ovo is a master regulator of germ cell transcription, Nejire is a chromatin-binding protein known to stabilize open, active chromatin, and Maf-S is a transcription factor essential for GSC self-renewal in the testis. In contrast, the subtype 3 network includes factors that are maternally inherited and necessary for embryogenesis [i.e. Pdm3, Nubbin (Nub), Distalless (Dll), and Luna (homolog of mammalian KLF6); Ng et al., 1995; Özel et al., 2022; Plavicki et al., 2016; Weber et al., 2014]. Thus, subtype 3 likely represents the earliest transcription of maternally expressed genes during germ cell differentiation.

SCENIC analysis also predicted regulation of germ cell differentiation by the transcription factor encoded by *longitudinals lacking* (*lola*; Fig. 7A). The *lola* gene locus is complex, spanning over 61 kb and known to encode at least 20 different proteins. Lola is required for gonad morphogenesis, stem cell maintenance, and differentiation (Bass et al., 2007; Davies et al., 2013; Silva et al., 2016; Zhao et al., 2022). SCENIC analysis predicted high Lola regulon activity in subtypes 0-2 (Fig. 7A). To validate the computational analysis, we used an antibody against the Lola-DE isoform and available fluorescently tagged transcriptional reporter lines to probe expression in wild-type germaria (Fig. 7C-E). In contrast to pan-Lola antisera, which detect protein ubiquitously in the germarium (Zhao et al., 2022), anti-Lola-DE antisera detected a nuclear protein specifically enriched in GSCs and cystoblasts (Fig. 7C). Intriguingly, however, C-terminally tagged fluorescent reporters of the *lola-DE* and *lola-I* isoforms were not expressed in undifferentiated cells and instead localized to differentiated 16-cell cyst nuclei (Fig. 7D,E). These data open the possibility that Lola influences gene expression programs during germ cell differentiation, and further highlight the complexity of gene regulation at the transcriptional and post-transcriptional level during the switch from stem cell to maternal factor expression. Notably, a *lola* paralog, *lola like* (*lolal*), is essential for cyst growth (Kotb et al., 2026). Together, these analyses suggest that BMP and ecdysone signaling converge on a common program that controls fate transitions in the germline (Fig. 7F).

## DISCUSSION

A key question in steroid hormone biology is how a single hormone elicits distinct cellular responses within the same tissue. In *Drosophila*, ecdysone signals through the ecdysone receptor, a heterodimeric nuclear receptor complex composed of EcR and Usp. Here, we demonstrate that germ cells are controlled autonomously by EcR and respond differently depending on differentiation state. We propose that chromatin state, high expression of the co-repressor Smr, and spatial positioning within a BMP-enriched niche allow EcR to direct a distinct transcriptional program in undifferentiated germ cells (Fig. 7F). Together with non-autonomous EcR signaling in somatic cells, EcR activity may establish a positive feedback loop that parallels BMP signaling to suppress differentiation. We postulate that, as Smr levels decline in dividing cysts, EcR becomes de-repressed and, in the presence of increasing ecdysone supplied by escort cells, activates a transcriptional cascade of nuclear receptors and other factors that promote cyst growth, germ cell maturation, and maternal mRNA accumulation. Thus, our findings establish the ovary as a tractable system for dissecting how a steroid hormone generates cell type-specific outcomes.

### Defining an ecdysone-responsive transcriptional cascade in ovarian cells

Our current understanding of the ecdysone response in *Drosophila* cells was guided by the Ashburner model, a simplified framework for ligand-receptor interactions defined in larval salivary glands (Morrow and Mirth, 2024; Thummel, 1996; Uyehara and McKay, 2019; Yamanaka et al., 2013). In this model, low ecdysone titers induce a small set of ‘early’ EcR target genes, including *Eip75B*, *br*, *ftz-f1*, and *Eip93F,* while high ecdysone titers allow early gene products to repress their own transcription and transactivate more than 100 ‘late’ genes, thought to be tissue specific (Thummel, 1996). Most refinements of this model examined somatic cells, leaving open the possibility that germ cells, which have specialized transcriptional and post-transcriptional mechanisms to regulate gene expression, respond to the hormone by unique mechanisms. Moreover, the model does not explain how individual cells make different responses to the same hormone, or how receptor titer, ligand availability, and chromatin state shape those responses.

Our data extend the Ashburner model to a steady-state, hormone-producing tissue rather than a pulse-driven system. In adult ovaries, late-stage ovarian cells (i.e. oogenesis stages 8-10, outside of the germarium) produce high ecdysone titers and express abundant EcR and Usp. In contrast, undifferentiated cells express lower receptor levels (Rust et al., 2020; Slaidina et al., 2021), and escort cells likely provide low-titer hormone production in the germarium (Fig. S4) (Shi et al., 2021). Although we did not quantify endogenous isoform-specific receptor abundance, our data suggest receptor titer, rather than isoform, primarily determines response timing. We also find that the differentiation status of ovarian cells is likely a defining factor for hormonal cell type specificity. Cells within the germarium microenvironment vary in developmental plasticity, including tissue-resident stem cells, differentiating cells, and terminally specified cells of multiple lineages. Accordingly, expression of the early-response genes varies dependent on cell type, as *Eip75B* and *ftz-f1* predominate in GSCs and differentiating germ cells, while *br* and *Eip93F* are enriched in germarium somatic cells. Because chromatin organization strongly influences cellular plasticity, chromatin state likely acts as a key modifier of ecdysone responsiveness. GSCs possess open euchromatin, while immediate daughters rapidly accumulate heterochromatin (Pang et al., 2023). EcR preferentially binds open chromatin, and ecdysone-responsive genes or co-regulators may further modulate chromatin accessibility (Badenhorst et al., 2005; Carbonell et al., 2013; Kimura et al., 2008; Sedkov et al., 2003; Uyehara et al., 2022). Future studies comparing the chromatin state in individual cells of the germarium will provide further insight about how cells sense and respond in unique ways to the same hormone.

### Emerging questions about the control of hormone and receptor titer in the germline

Our findings support a model in which EcR transitions from a transcriptional repressor to an activator during germline differentiation. Several mechanisms may underlie this switch. First, low local ecdysone levels may maintain EcR in a repressive state, consistent with evidence that the EcR/Usp complex represses transcription at low ligand concentrations and autoregulates its own expression (Morrow and Mirth, 2024; Nogueira Alves et al., 2022; Perez-Mockus et al., 2023). Because early germ cells do not appear to synthesize their own ecdysone (Fig. S4) (Shi et al., 2021), anterior escort cells likely provide limited ligand. As follicle cells from first-wave egg chambers grow, both ovarian and circulating ecdysone concentration likely increase. Thus, EcR may repress itself in undifferentiated germ cells. Second, co-regulator availability likely modulates EcR output. The co-activator Taiman (Tai) is expressed at low levels and is dispensable in undifferentiated germ cells (Ables and Drummond-Barbosa, 2010), whereas the co-repressor Smr (analogous to mammalian nuclear receptor co-repressor 2 or NCOR2) is highly expressed in GSCs and early cysts but declines as cysts divide (Fig. S10). Rising ecdysone levels combined with reduced Smr expression may relieve EcR-mediated repression in later cysts. Third, sequential activation of EcR-dependent transcription factors may constrain EcR activity through feedback regulation. We observed upregulation of *Eip75B*, *Eip78C*, and *ftz-f1* in dividing cysts (Fig. 6). *Eip75B* limits GSC self-renewal (Fig. S9) (Ables and Drummond-Barbosa, 2010), and *ftz-f1* is required for both GSC maintenance and cyst division (Fig. 6). Both factors repress *EcR* expression in other contexts (Boulanger et al., 2011; Johnston et al., 2011). Since EcR and Ftz-f1 share few predicted transcriptional targets in cystoblasts (e.g. *bam^Δ86^* mutant cells), EcR likely acts as a self-limiting temporal switch rather than a sustained driver of transcription.

### The ovarian GSC microenvironment/niche as a unique model for studying the convergence of nuclear receptor signaling with other signaling pathways

The ability of stem cells to preserve tissue structure and optimize organ function relies on their ability to sense and respond to tissue-level and organism-level regenerative cues (Das et al., 2020; de Morree and Rando, 2023). A fundamental feature of this response is the organization of stem cells within tissue microenvironments (niches) that dictate their accessibility to local and systemic signals (Comazzetto et al., 2021; Xin et al., 2016). This is well illustrated in the *Drosophila* ovary, where germline and follicle stem cells are centered at a nexus of diffusible signals (TGFβ/BMP, Wnt, EGFR, and Hh) that influence their fate (Losick et al., 2011). Yet how endocrine signals are received by stem cells in non-vascularized areas, and whether these cues are interpreted more specifically than would a general pro-proliferative or pro-survival signal, has been more difficult to decipher. Our study sheds light on this complex problem and offers an elegant model to understand hormonal regulation of stem cells in the context of the other signals that influence cellular activity. Our results highlight the remarkable cell-specific effects of steroid hormone signaling within a single tissue, illustrating how one hormone can drive dramatically different transcriptional outcomes in adjacent cells. Although steroid hormones are typically considered long-range signals, we propose that local sources of steroid production or areas of receptor turnover or inactivation may likewise influence tissue-resident stem cell activity. Understanding how tissue microenvironments modify, shield, or concentrate steroid hormone titer or response may provide new avenues for modifying stem cell activity *in vivo*.

## MATERIALS AND METHODS

### *Drosophila* strains and culture conditions

Flies were maintained at 22-25°C on a standard medium containing cornmeal, molasses, yeast, and agar (Nutrifly MF; Genesee Scientific). For all experiments, unless otherwise noted, flies were collected within 24 h of eclosion and maintained at 25°C on standard medium supplemented with wet yeast paste for 5-6 days (changed daily) prior to ovary dissection. For *dpp* temperature-sensitive mutants, crosses were set at 22°C and maintained at 18°C, progeny were collected within 24 h of eclosion, and progeny were transferred to 25°C and fed for 5 days prior to dissection. Germline expression of transgenes or dsRNA hairpins was facilitated by expressing the germline-specific *nos-GAL4::VP16-nos.UTR* [referred to throughout as *nos-Gal4::VP16*; Bloomington *Drosophila* Stock Center (BDSC) #4937] (Rørth, 1998; Van Doren et al., 1998). Driver expression was confirmed using *UASp-tub84BGFP* (BDSC #7373) (Grieder et al., 2000) or *UASp-lacZ* (BDSC #98113) (Rørth, 1998). Females carrying *nos-Gal4::VP16* and *UASp-lacZ* were used as controls. A complete list of all transgenes used in this study can be found in Table S1.

### Construction and validation of *UAS-EcR^RNAi^* transgenes

*UAS-EcR^RNAi^* lines were generated in *pVALIUM22* transgenes as described (www.flyrnai.org/TRiP-HOME.html) (Ni et al., 2011) for maximum germline efficiency. *UAS-EcR^RNAi2^* and *UAS-EcR^RNAi3^* target an *EcR* coding exon common to all isoforms, using the same hairpin sequence as HMC03114 (5ʹ-CCGAGATGTGTTTCTCACTAA-3ʹ) and HMJ22371 (5ʹ-CTGCGATGCGAAGAAGAGCAA-3ʹ), respectively. *UAS-EcR^RNAi1^* was designed against the *EcR-A* isoform via the Designer of Small Interfering RNA tool (https://biodev.cea.fr/DSIR/DSIR.html) using default settings (21 nt siRNA; score threshold 90; 5ʹ-CAACAGTAGCTACGCTAGATC-3ʹ); however, our analyses failed to confirm that the resulting dsRNA is specific to *EcR-A* (Fig. S1A-C). Primers were designed according to the Harvard Transgenic RNAi Project (TRiP) recommendations, annealed to form double-stranded oligonucleotides, and ligated into *EcoRI-HF*/*NheI-HF*-digested *pVALIUM22*. Transgenic flies were generated by phiC31 site-specific integrase into the *attP2* site on the third chromosome (Bestgene). To validate *EcR* mRNA depletion in *UAS-EcR^RNAi^* lines, primers were designed to span the common coding region (exons 3 and 4) shared by *EcR-A* and *EcR-B1* isoforms, minimizing amplification of genomic DNA. All primers are listed in Table S1.

To assess RNAi efficacy, germline knockdown was facilitated by *nos-GAL4::VP16* and RNA extraction was performed by dissecting 15 pairs of ovaries from females fed wet yeast paste for 5 days after eclosion. Ovaries were dissected in Invitrogen RNAlater (Thermo Fisher Scientific/Invitrogen) solution and transferred to 1.5 ml tubes containing RNAlater until the time of extraction. Whole-ovary RNA extraction was performed using the RNAqueous-4PCR RNA Isolation kit (Thermo Fisher Scientific/Invitrogen). RNA concentration and purity were determined using the Agilent Bioanalyzer RNA Pico assay and stored at −80°C. Genetic replicate samples were used to generate cDNA using iScript synthesis kit (Bio-Rad). Reverse transcription PCR amplification of generated cDNA was run at 25 cycles at a melting temperature of 57°C for three genetic replicates. PCR products were run on a 1.5% agarose gel to measure intensity levels, normalized to the ribosomal protein encoded by *RpL32*.

### Construction of *EcR* overexpression and dominant-negative transgenes

To generate *UASz*-*EcR* overexpression and dominant-negative constructs, the *EcR* coding region with or without nucleotide substitutions was synthesized into *pUC57* mini (GenScript) following the methods of Cherbas and colleagues (Cherbas et al., 2003; Hu et al., 2003), creating *pUC57-EcR-B1^ΔC655^*, *pUC57-EcR-B1^ΔC655-F645A^*, *pUC57-EcR-B1^ΔC655-W650A^*, and *pUC57-EcR-B1^ΔC655-F645A+W650A^* (referred to as *pUC57-EcR-B1^DBL^*). Likewise, the coding region for *EcR-A* was designed with sequences equivalent to *EcR-B1* (*pUC57-EcR-A^ΔC627^*). Coding regions were amplified from *pUC57* mini and cloned into the *BamHI* site of *pUASz-1.0* (DeLuca and Spradling, 2018) or a modified *pUASz-1.0* with *mCherry* in frame at the C- or N-terminus using InFusion Snap Assembly Cloning (Takara). *EcR-A* dominant-negative transgenes [*EcR-A^ΔC627-F617A^*, *EcR-A^ΔC627-W622A^*, and *EcR-A^ΔC627-F617A+W622A^* (referred to as *EcR-A^DBL^*)] were cloned by site-directed mutagenesis in *pUASz-EcR-A*. Plasmid sequences were verified via nanopore sequencing (Plasmidsaurus). Plasmid DNA was injected into embryos by BestGene and docked into the *attP40* locus on chromosome two by phiC31-mediated integration.

### Genetic mosaic generation

For genetic mosaic analyses with negative labeling using flippase (FLP)/FLP recognition target (FRT), we used the null allele *ftz-f1^ex7^* (BDSC #64338) on *FRT79D*. Other genetic tools are described in FlyBase version FB 2025_04 (Öztürk-Çolak et al., 2024). Genetic mosaics were generated by FLP/FRT-mediated recombination in 2- to 3-day-old females carrying a mutant allele in trans to a wild-type allele (linked to a *Ubi-nGFP* marker) on homologous FRT arms, and a *hs-FLP* transgene, as described (Laws and Drummond-Barbosa, 2015). For induction of transgene expression by FLP-out genetic recombination, we used *hsFLP; nos-FRT-STOP-FRT-Gal4, UASp-H4-eGFP* (a gift from Ting Xie, Hong Kong University of Science and Technology, Hong Kong) (Phipps et al., 2023). Flies were heat shocked at 37°C two times per day for 3 days and incubated at 25°C supplemented with wet yeast paste prior to dissection. Wild-type alleles were used for the generation of control (mock) mosaics.

### Immunofluorescence and microscopy

Ovaries were dissected, fixed, washed, and blocked as previously described (Ables et al., 2016; Hinnant et al., 2017; Williams and Ables, 2023). In the standard protocol, ovaries were dissected and teased apart in Grace's medium (Caisson Labs) and fixed in ice-cold 5.3% formaldehyde in Grace's medium at room temperature for 13 min. A modified protocol was also used for some antibodies, wherein ovaries were fixed with room temperature 5.8% formaldehyde in Grace's Insect Medium for 10 min at room temperature. Ovaries were washed extensively in phosphate-buffered saline (PBS, pH 7.4; Thermo Fisher Scientific) with 0.1% Triton X-100 (PBST), permeabilized in PBS with 0.5% Triton X-100, then blocked for 1-3 h in a blocking solution consisting of 5% bovine serum albumin (BSA; Sigma-Aldrich), 5% normal goat serum (MP Biomedicals) and PBST. Samples were incubated with primary antibodies in blocking solution at 4°C overnight. Samples were incubated with Alexa Fluor 488-,568-, or 633-conjugated goat-species specific secondary antibodies (Thermo Fisher Scientific; 1:200). A complete list of antibodies is listed in Table S1. Where appropriate, EdU was detected using Alexa Fluor 594 or 647 via Click-It chemistry following the manufacturer's recommendations (Thermo Fisher Scientific). Ovaries were counterstained with DAPI (Sigma-Aldrich, 1:1000 in PBS). Ovaries were then mounted in 90% glycerol containing 20.0 μg/ml N-propyl gallate (Sigma-Aldrich). Data were collected using a Zeiss LSM 700 laser scanning confocal microscope equipped with a 63× plan apo objective. Images were analyzed using Zen Black software and images were minimally and equally enhanced via histogram using Zen and Adobe Photoshop CC.

### Germ cell analyses and statistics

GSCs were identified based on the juxtaposition of their fusomes (at any morphology) to the junction with adjacent cap cells (Ables and Drummond-Barbosa, 2010; Villa-Fombuena et al., 2021). Germ cell differentiation was scored as the number of germaria with a blocked differentiation phenotype, compared to controls and subjected to Fisher's exact test analyses using Prism (GraphPad). In *FLP/FRT* mosaics, GSC loss was measured as the percentage of total germaria analyzed that had at least one GFP-negative GSC, and subjected to Fisher's exact test (Laws and Drummond-Barbosa, 2015). In RNAi progeny, GSC loss was measured as the average number of GSCs present over three timepoints. Results were subjected to Student's two-tailed, unpaired *t*-test at each timepoint using Microsoft Excel and Prism.

### scRNA-seq and analyses

Using method A (Slaidina et al., 2021), 100 ovaries from each genotype were dissected in Grace's insect medium and disassociated in a solution containing 0.5% type 1 collagenase and 1% trypsin in PBS solution for 15 min using vigorous pipetting every few minutes. Disassociation was stopped by adding Grace's insect medium with 10% fetal bovine serum. Suspended cells were strained through a Flowmi 40 μm filter tip (Sigma-Aldrich) and then centrifuged at 2.4 ***g*** for 3 min. The supernatant was decanted, cells were resuspended in 0.04% BSA in PBS and centrifuged again at 2.4 ***g*** for 3 min. The final suspension was in Grace's insect medium and the sample was preserved on ice. Live/dead cell count was performed on the Invitrogen Countess 3FL Cell Counter. 1.70×10^6^ cells from the *nos-Gal4>UASp-tkv^ACT^* sample were collected (89% viability) and 1.17×10^7^ cells from the *nos-Gal4>UASz-EcR-B1* sample were collected (95% viability).

Libraries were constructed using 10x Genomics Chromium single cell 3′ V3 kit according to the manufacturer's protocol. Sequencing of the pooled libraries was performed on the NextSeq 2000 platform (Illumina). For the *EcR-B1* sample, 5741 cells were sequenced with a total of 136,771,995 reads [an average of 23,799 reads per cell; 96.70% valid barcodes; 74.6% mapped confidently to the genome; 4112 median unique molecular identifier (UMI) counts per cell]. For the *tkv^ACT^* sample, 7043 cells were sequenced with a total of 116,346,480 reads (an average of 16,519 per cell; 97.40% valid barcodes; 96.8% mapped confidently to the genome; 6934 median UMI counts per cell). Data can be accessed from the NCBI Gene Expression Omnibus (GEO) database GSE309824.

Raw sequencing reads in FASTQ files were obtained using cellranger (v7.2.0) mkfastq (10x Genomics). The alignment to *Drosophila melanogaster* reference genome BDGF6.46 (Ensembl) and the followed quantification was conducted using cellranger count. Wild-type ovary raw scRNA-seq data were downloaded from the NCBI GEO database GSE162192 (Slaidina et al., 2021). *bam^Δ86^* raw sequencing data are from Sun et al. (2025 preprint) and can be accessed at the Genome Sequence Archive (CRA028191), from which 6800 single cells were randomly selected for analysis (to keep the total number of cells comparable to those from *EcR-B1* and *tkv^ACT^* samples).

Subsequently, the Seurat (v5.1.0) package (https://github.com/satijalab/seurat) in RStudio (Build 369) with R (v4.4.1) was used for data analysis. Briefly, single cells were filtered with nFeature_RNA (more than 1000 but less than 6000). Doublets or multiplets were identified by DoubletFinder (v2.0.4; https://github.com/chris-mcginnis-ucsf/DoubletFinder), mainly in differentiated germ cells of control samples (4-cell, 8-cell, and 16-cell cysts) and were thus not eliminated. Batch effects were corrected using Harmony (v1.2.0; https://github.com/immunogenomics/harmony). Gene expression data were then aggregated, normalized, and variance stabilized with SCTransform (Seurat)-regularized negative binominal regression. Dimensionality reduction was performed by principal component analysis (PCA) and uniform manifold approximation and projection (UMAP) embedding (dims=1:30) with RunPCA and RunUMAP, respectively. Cell clusters were subsequently identified with FindNeighbors (dims=1:30) and FindClusters (resolution=0.2 for initial clustering and 0.5 for refined clustering); and annotated based on expression of cell type-specific gene biomarkers. Differentially expressed biomarkers among cell types were assessed with FindAllMarkers or FindMarkers for genes detected in at least 10% of cells using a log(fold change) threshold at 0.25. Bonferroni-adjusted *P*-values were used to determine significance at a false discovery rate <0.05.

The aggregated data set of wild-type (GSE162192), *bam^Δ86^* (CRA028191), *EcR-B1* and *tkv^ACT^* (GSE309824) were visualized using the 10x Loupe Browser platform. The aggregated data of both samples pipeline-generated 16 clusters. When measuring differentially expressed genes between clusters and groups, log_2_(fold change) is the ratio of the normalized mean gene UMI counts in the cluster relative to all other clusters. *P*-values were adjusted using the Benjamini–Hochberg correction for multiple tests.

## Supplementary Material



10.1242/develop.205460_sup1 Supplementary information

Table S2. Combined single cell mRNA expression from four genotypes (wild-type, *EcR-B1*, *tkv^ACT^*, and *bam^Δ86^*), per cell cluster. Gene expression data used to assign cell type identities to each cluster. Relates to Figures 3-5 and 6A.

Table S3. Combined single cell mRNA expression from four genotypes (wild-type, *EcR-B1*, *tkv^ACT^*, and *bam^Δ86^*), including undifferentiated germ cells only. Gene expression data used to assign cell type identities to each cluster. Relates to Figures 3-5 and 6A.

Table S4. Results from SCENIC analyses, predicting transcription factors regulating gene expression in each undifferentiated cell cluster. Relates to Figures 6H and 7A.
