# Supplementary material for: Ecdysone receptor autonomously controls germ cell differentiation in the Drosophila ovary
Source: Development. 2026 Jul 29;153(14):dev205460. doi: 10.1242/dev.205460 (PMC13489555; doi:10.1242/dev.205460)
Supplement: Supplementary file 1 [file develop-153-205460-s1.pdf]

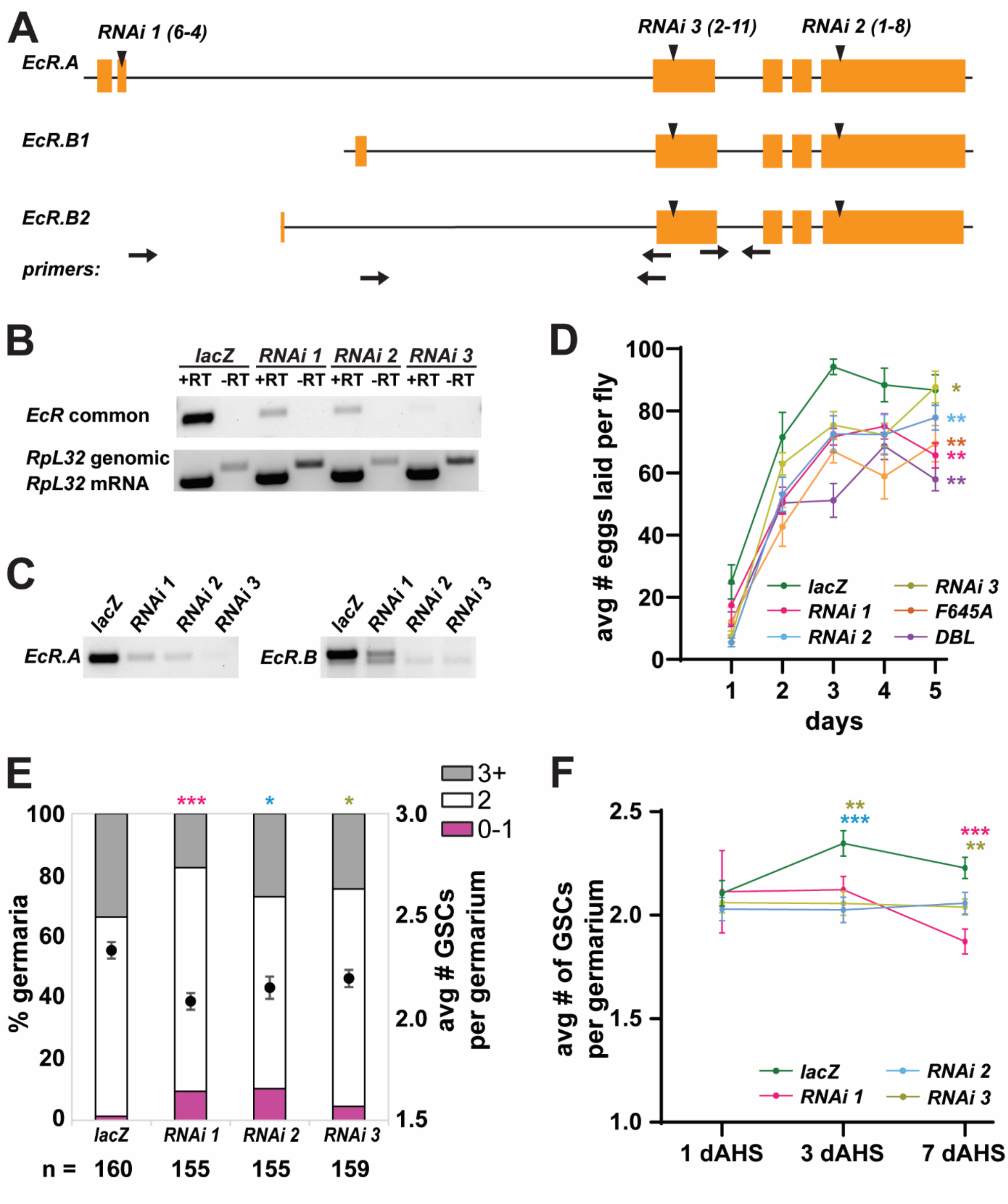

Fig. S1. Validation of germline compatible *UAS-EcR*^{RNAi} transgenes.

(A) The *EcR* gene locus encodes three major mRNA isoforms (*EcR-A, EcR-B1, and EcR-B2*). Arrowheads indicate gene region targeted by RNAi hairpins; arrows indicate gene regions amplified by RT-PCR. (B-C) Semi-quantitative RT-PCR analysis of RNAi efficiency (B, using primers commons to all isoforms) and isoform specificity (C, using primers specific for *EcR-A* or *EcR-B*) in whole ovaries depleted for *EcR* via RNAi hairpins under control of *nos-Gal4::VP16*. Ovary size and egg chamber composition were roughly equivalent between wild

Development • Supplementary information

type and experimental samples. Since wild type ovaries represent the level of *EcR* mRNA that is equivalent to somatic mRNA plus germ cell mRNA, *EcR* mRNA could be detected in all *nos>EcR RNAi* experimental samples at plateau phase (32-35 cycles). Samples in the gel image presented were collected at 24 PCR cycles, which was determined to be within the linear range of amplification for control samples (labeled "lacZ") normalized to the ribosomal protein-coding gene *RpL32*. *EcR* mRNA was readily detected in the wild type sample, but greatly reduced in the *nos>EcR RNAi* experimental samples, confirming that less *EcR* mRNA is made in the germline in the presence of the RNAi. "RT" indicates samples with (+RT) or without (-RT) reverse transcriptase. Note that even though EcR^{RNAi1} was designed to target the *EcR-A* isoform, it depletes *EcR* mRNA in a non-specific manner, likely due to self-regulation of EcR (see (Karim and Thummel, 1992; Romani et al., 2016). (D) Comparison of the daily number of eggs laid per fly between control (lacZ) and *EcR*-manipulated (EcR^{RNAi1}, EcR^{RNAi2}, EcR^{RNAi3}, EcR^{F645A}, or EcR^{DBL}) females. Error bars represent s.e.m.; **p*<0.05, ***p*<0.01, Student's two-tailed t-test. Note that flies from each transgenic background produced fewer eggs than control flies, but were not statistically different from each other. (E) Frequencies of germaria containing zero or one, two, or three or more GSCs (left y axis), and average number of GSCs per germarium (right y axis) in in *nos-Gal4::VP16>UASp-GFP::tubulin* control and EcR^{RNAi1}, EcR^{RNAi2}, or EcR^{RNAi3} ovaries at six days after eclosion (relates to Figure 1B-D). Number of germaria analyzed is below genotypes. Error bars represent s.e.m.; **p*<0.01, ***p*<0.001, ****p*<0.0001; Student's two-tailed t-test. (F) Quantification of GSCs per germarium in *hsFLP;nos-STOP-Gal4* control or *nos-FLP>*EcR^{RNAi1}, EcR^{RNAi2}, or EcR^{RNAi3} (relates to Figure 1E-G). Quantification performed at 1, 3, and 7 days after heat shock (dAHS); $n > 72$ germaria per genotype per timepoint; error bars represent s.e.m., ***p*<0.001, ****p*<0.0001, Student's two-tailed t-test. Note that the control and EcR^{RNAi1} data are the same as in Figure 1G.

Development • Supplementary information

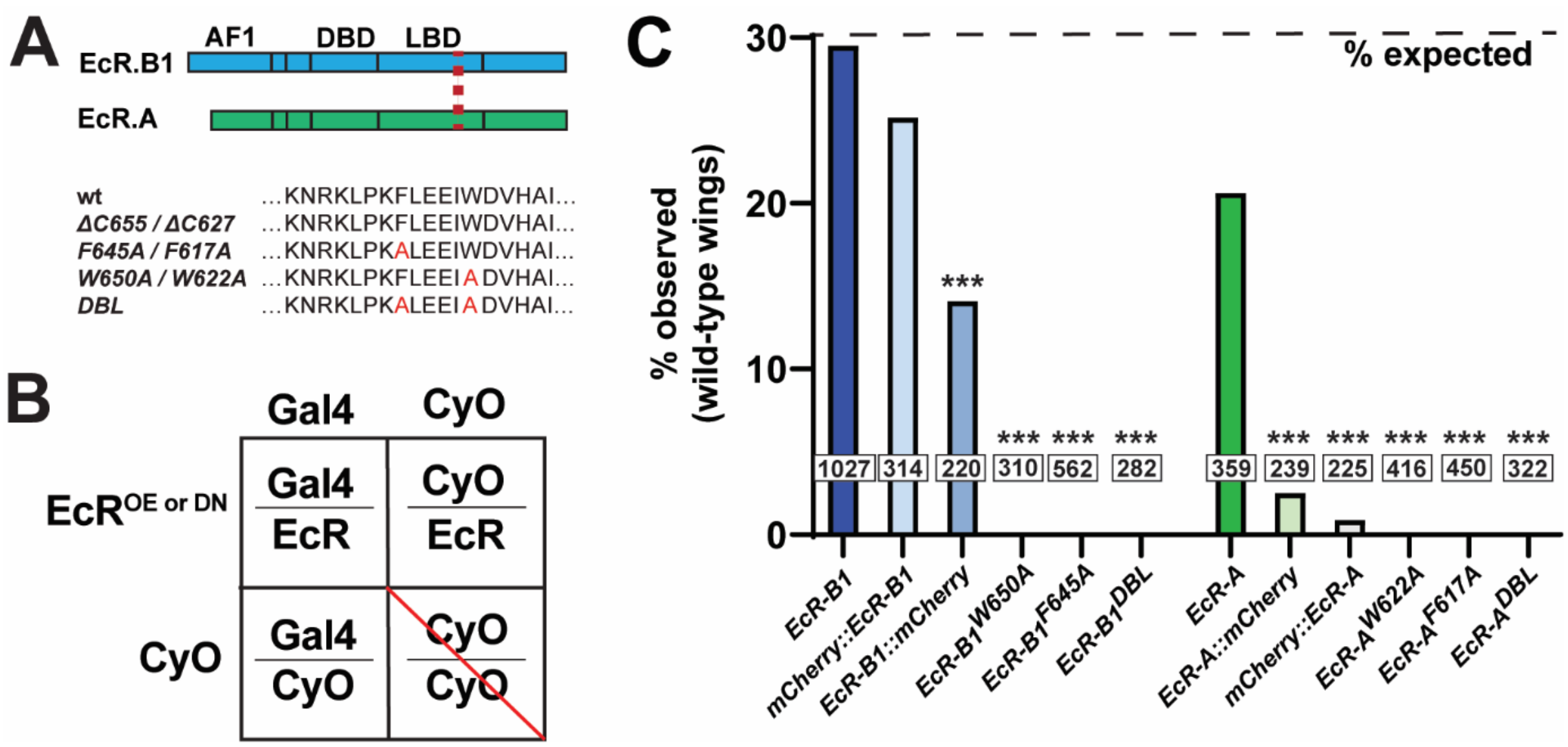

Fig. S2. Driving *UASz-EcR-A* and *UASz-EcR-B1* in all somatic cells induces larval lethality. (A) Schematic comparison of EcR-B1 and EcR-A proteins, indicating the DNA binding domain (DBD) and ligand binding domain (LBD). Red text indicates amino acid substitutions in the LBD, listed for wildtype and dominant-negative transgenes below. This sequence is identical between EcR-B1 and EcR-A, but has different nomenclature due to the overall length of the proteins. (B-C) *UASz-EcR* transgene efficacy was evaluated by lethality phenotypes under the control of *actin-Gal4*. Mendelian inheritance (B) predicts ~30% expected progeny should carry both *actin-Gal4* and the *UASz-EcR* transgene (dotted line in C). Over-expression of *EcR-B1* or *EcR-A* slightly reduced survival, while expression of the dominant-negative transgenes resulted in nearly complete lethality (C), consistent with prior studies. Numbers in boxes indicate the number of flies scored. ***p<0.0001, Chi-squared test.

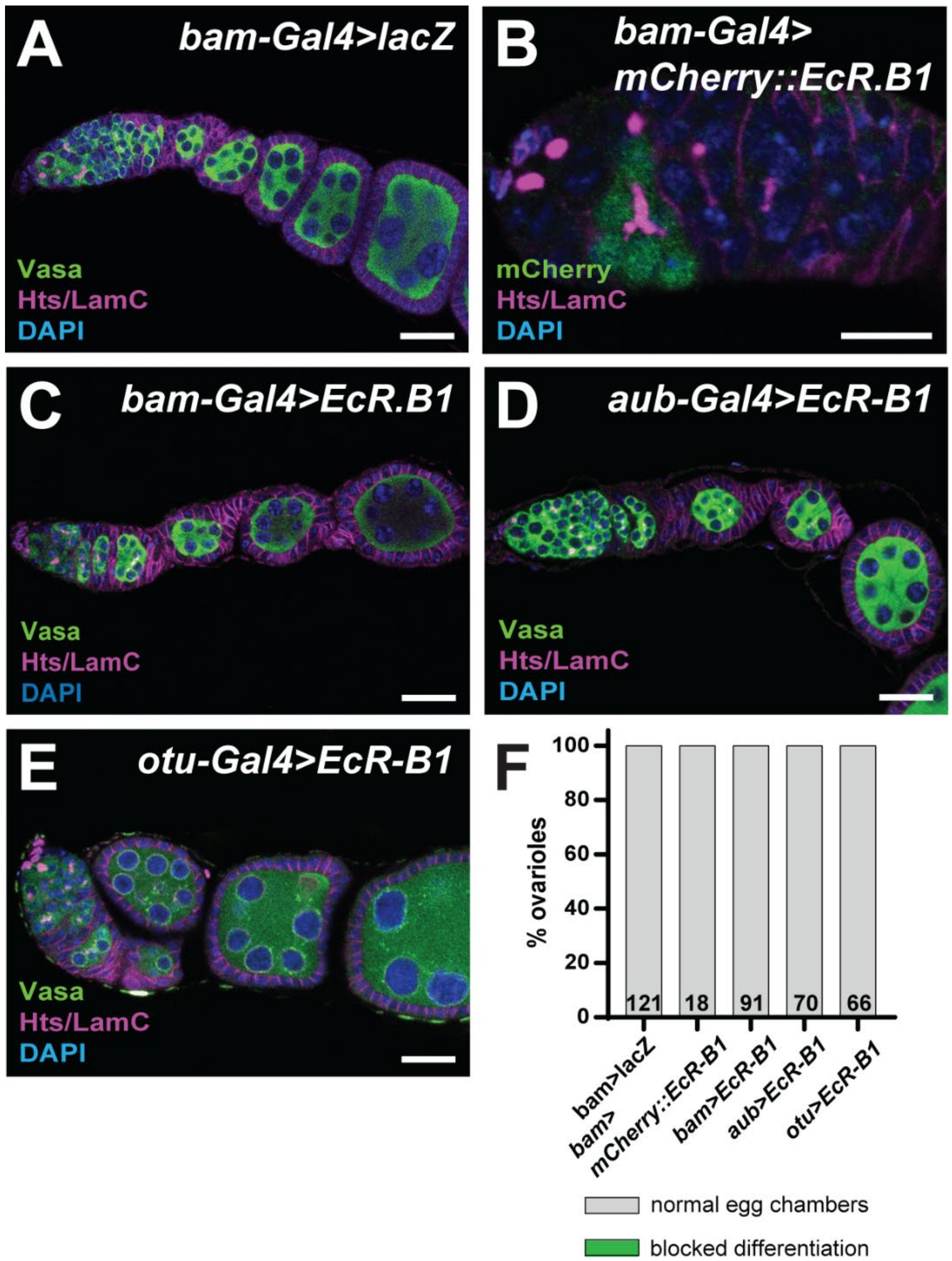

Fig. S3. ***UASz-EcR-B1*** **expression in dividing cysts is not sufficient to block germ cell differentiation. (A-E)** Representative images (A-E) and quantification (F) of *3x-Bam-Gal4* driving *UASp-lacZ* alone (A, control) or with *UASz-mCherry::EcR-B1* (B) or *UASz-EcR-B1* (C), or from *aub-Gal4* (D) or *otu-Gal4::VP16* (E) driving *UASz-EcR-B1* germaria immunostained for Vasa (green, germ cells, A,C-E) or mCherry (green, transgene expression, B), Hts (magenta; fusomes and follicle cells), LamC (magenta; cap cells and stalk cells), and DAPI (nuclei). In contrast to *nos-Gal4::VP16* (see Figure 2), over-expression of *EcR-B1* in cystoblasts or dividing cysts did not block germ cell differentiation. Bars in (F) represent the percentage of ovarioles displaying a block to germ cell differentiation; number in bars indicates the number of ovarioles scored, ~5 days after eclosion. ***p<0.001, compared to control; Fisher's exact t-test.

Development • Supplementary information

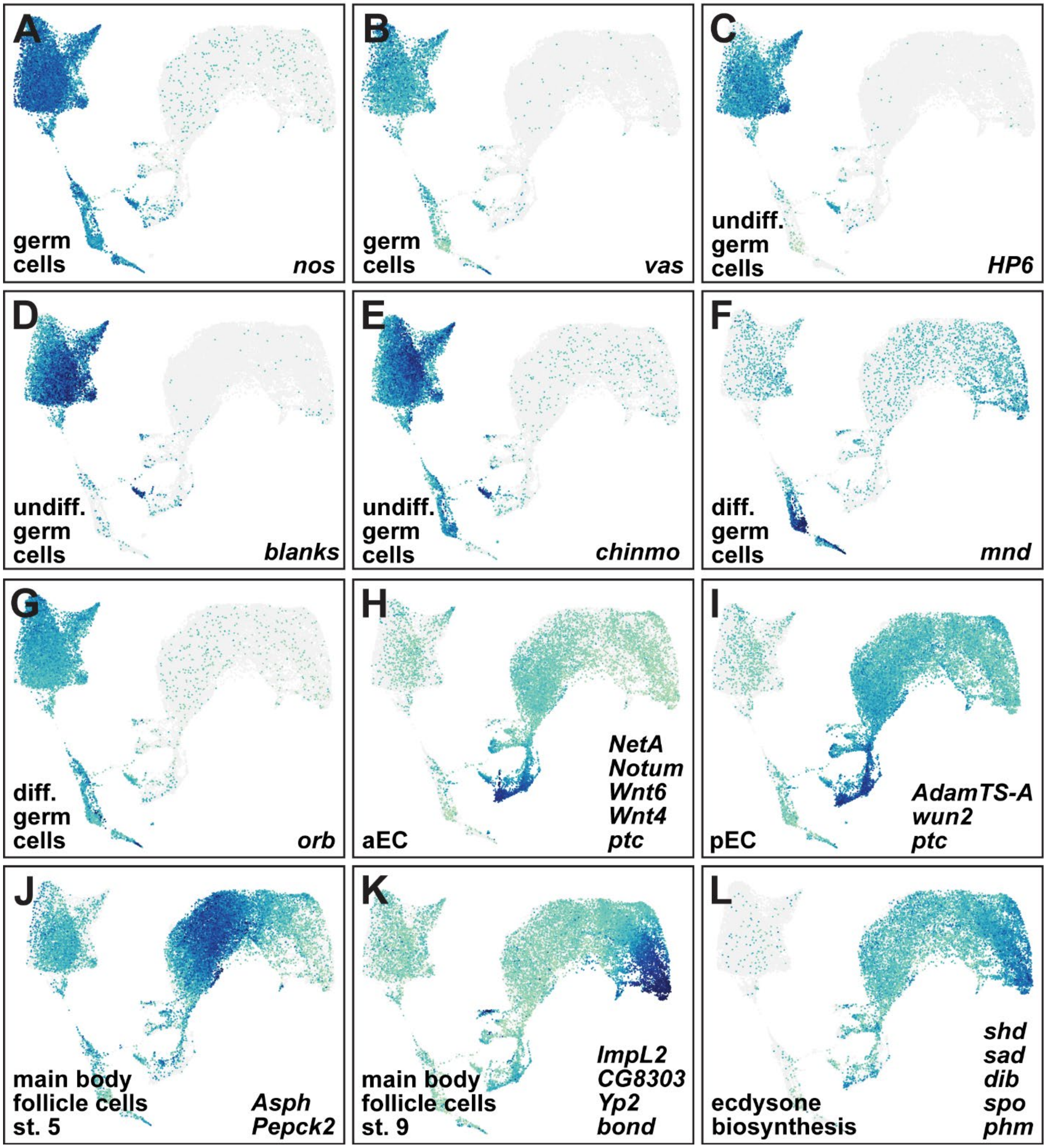

Fig. S4. Expression of cell type-specific genes distinguishes germline and somatic cell clusters. Representative expression level UMAPs of all cells merged from wildtype, *nos-Gal4::VP16>UASz-EcR-B1*, *nos-Gal4::VP16>UASp-tkv*^{ACT}, and *bam*$^{\Delta 86}$ ovaries, visualizing: the germ cell markers *nos* (A) and *vasa* (B); the undifferentiated germ cell markers *HP6* (C), *blanks* (D) and *chinmo* (E); the differentiated germ cell markers *mnd* (F) and *orb* (G); the anterior escort cell markers *NetA*, *Notum*, *Wnt4*, *Wnt6*, and *ptc* (H); the posterior escort cell markers *AdamTS-A*, *wun2*, and *ptc* (I); the main body follicle cell markers *Asph* and *Pepck2* (J; stage 5 egg chambers) or *ImpL2*, *CG8303*, *Yp2*, and *bond* (K; stage 9 egg chambers); and the ecdysone biosynthesis genes *shd*, *sad*, *dib*, *spo* and *phm* (L).

Development • Supplementary information

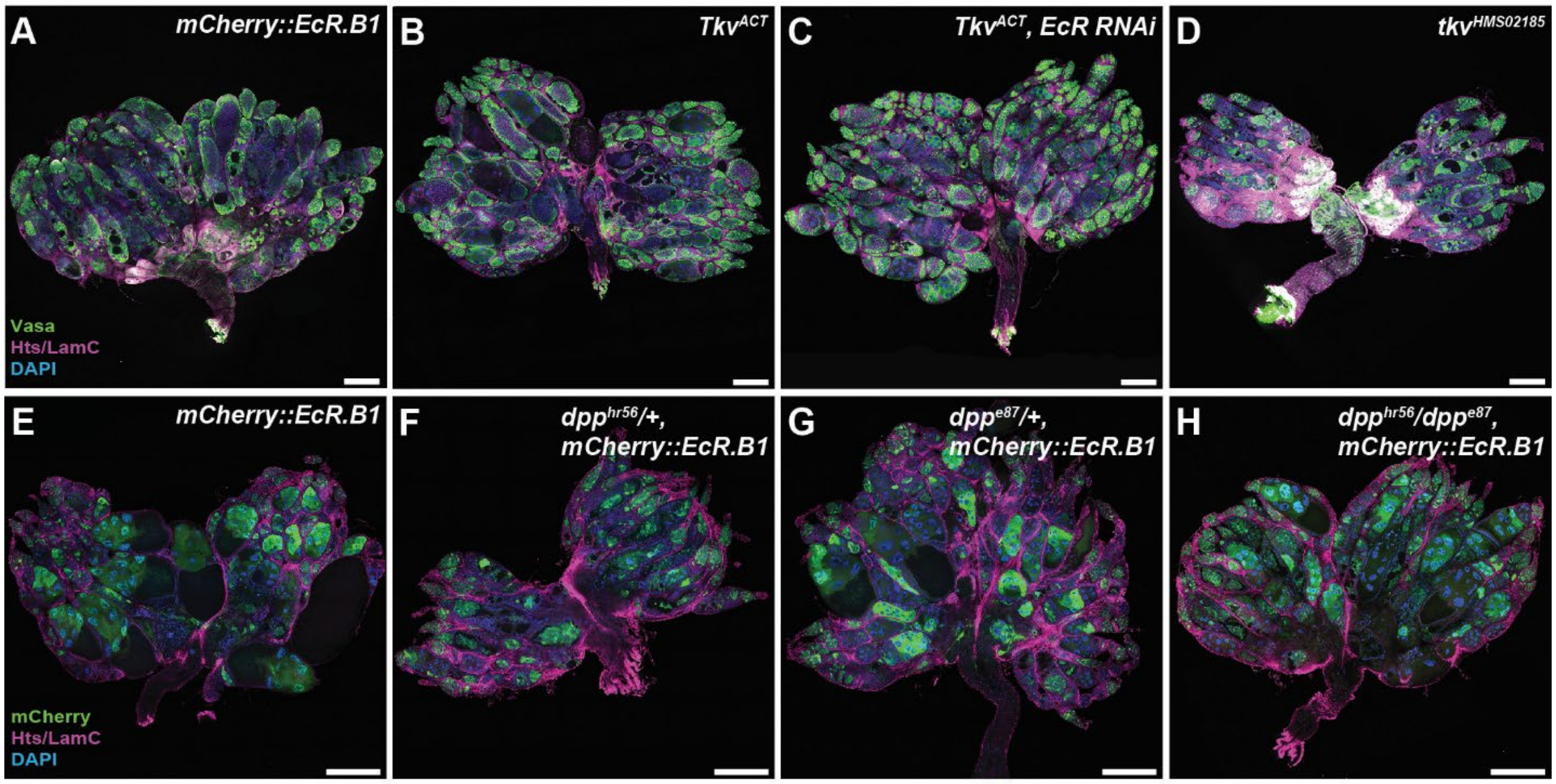

Fig. S5. Depletion of *tkv* or *dpp* is not sufficient to rescue germ cell differentiation in *UASz-EcR.B1*-expressing cells. (A-B,D-E) Over-expression of *mCherry::EcR* (A,E) or *tkv*^{ACT} (B), or knock-down of *tkv* by RNAi (D) in germ cells produces small ovaries that lack mature egg chambers. Transgenic ovaries are about one-fourth the size of normal ovaries, and flies are sterile. (C) Knock-down of EcR in the *tkv*^{ACT} produces equivalent phenotypes as over-expression of *tkv*^{ACT} alone. Similarly, reduction of one copy (F-G) or two copies (H) of *dpp* in *EcR* over-expressing germ cells produces equivalent phenotypes as over-expression of *EcR* alone (E). Ovaries were immunostained for Vasa (green, germ cells, A-D) or mCherry (green, transgene expression, E-H), Hts (magenta; fusomes and follicle cells), LamC (magenta; cap cells and stalk cells), and DAPI (nuclei). Scale bars represent 50µm.

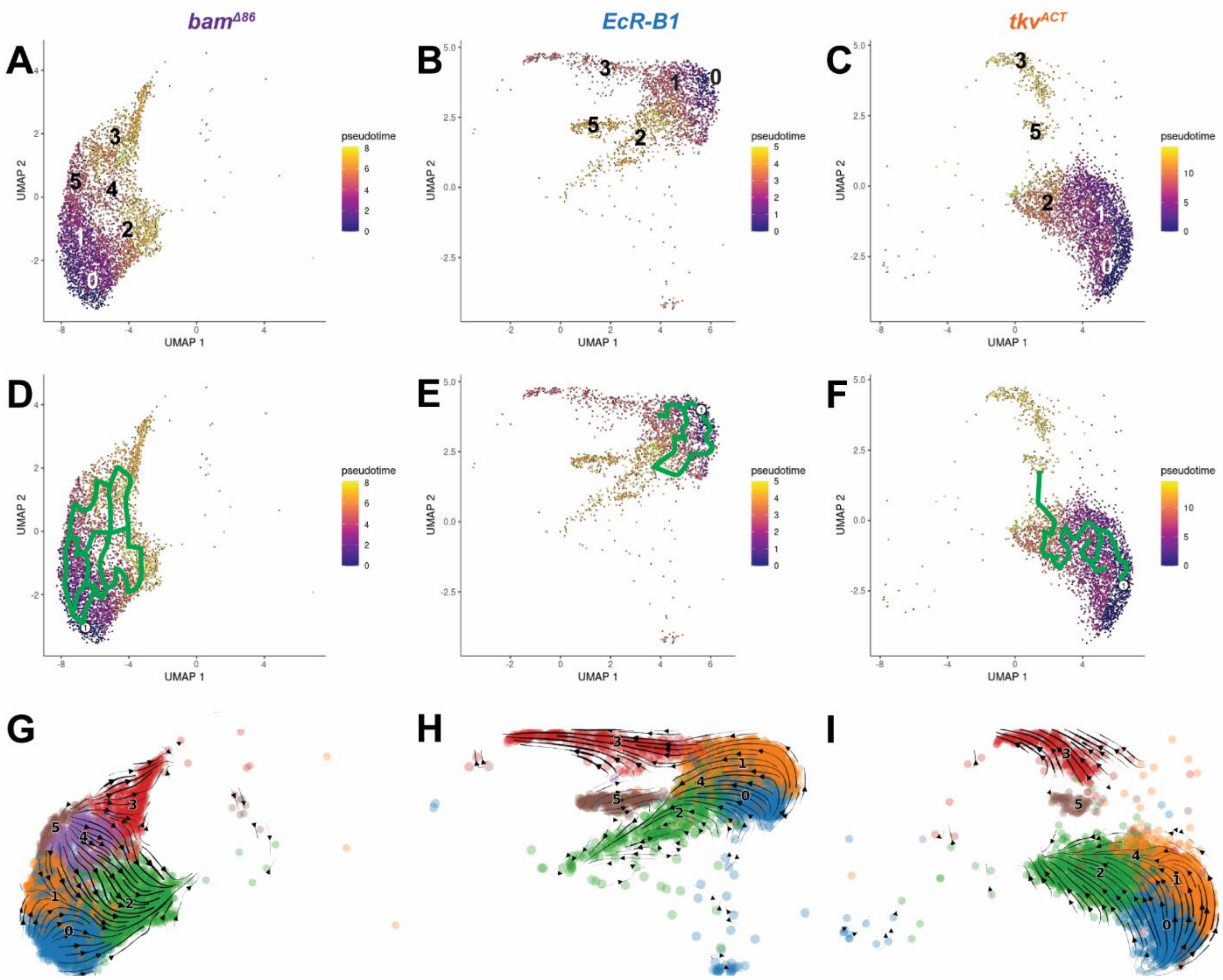

Fig. S6. Pseudotime analysis and single-cell velocity reveal stages of differentiation. (A-F) Pseudotime analysis of undifferentiated germ cells from (A) *bam*$^{\Delta 86}$, (B) *EcR-B1*, and (C) *tkv*^{ACT} ovaries. The lighter the color of the cells, the more differentiated they are. In panels D-F, the estimated trajectory of differentiation is overlayed in green. (G-I) Single cell velocity analysis of (D) *bam*$^{\Delta 86}$, (E) *EcR-B1*, and (F) *tkv*^{ACT}. Undifferentiated germ cell subtype 0 is in blue, 1 in orange, 2 in green, 3 in red, 4 in purple, and 5 in brown.

Development • Supplementary information

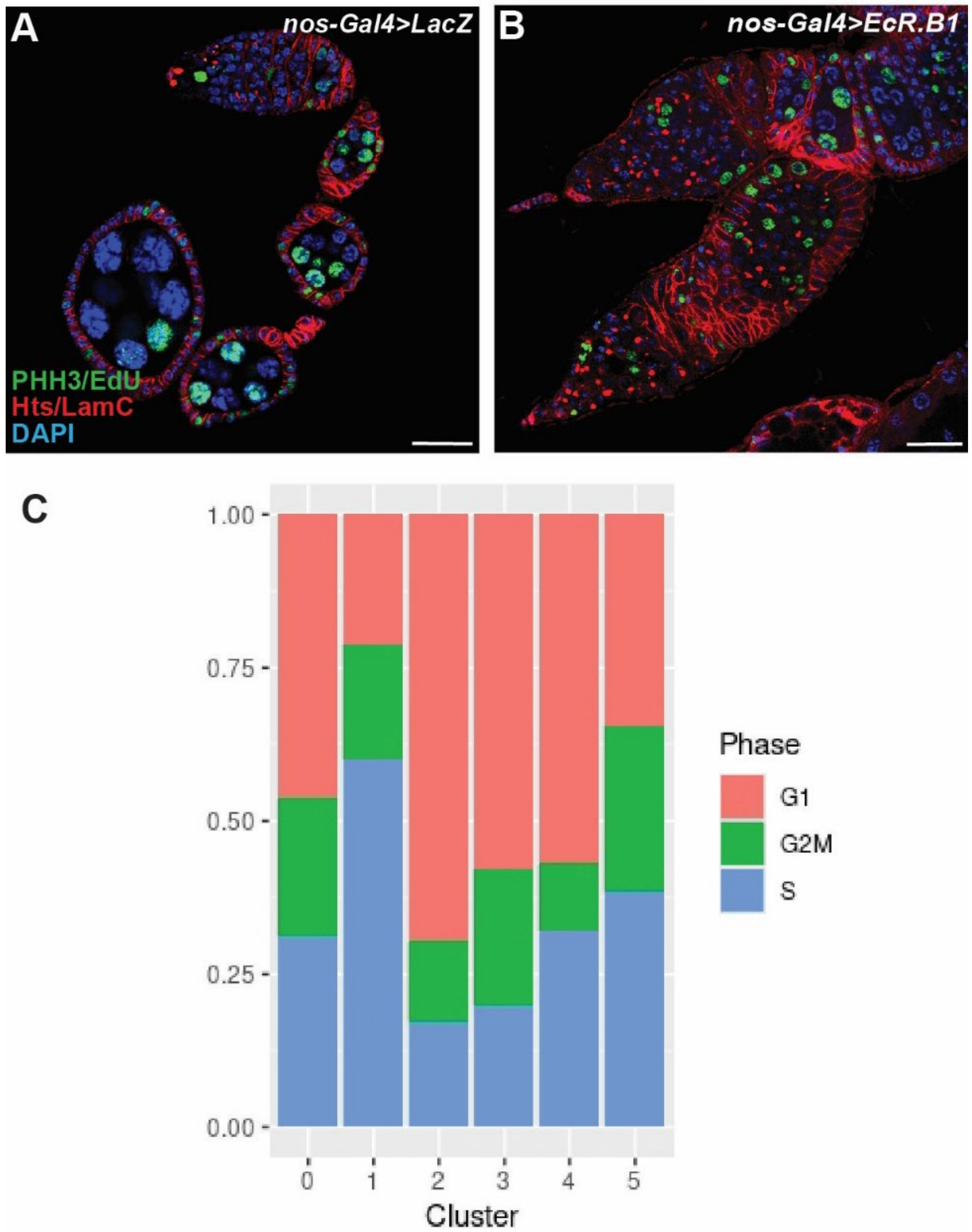

Fig. S7. *EcR* over-expressing germ cells are proliferative. (A-B) Control (A) and *EcR* over-expression (B) ovaries immunostained for the active cell cycle markers phosphorylated Histone H3 (PHH3) and EdU (green), Hts (magenta; fusomes and follicle cells), LamC (magenta; cap cells and stalk cells), and DAPI (nuclei). Scale bar = 20 µM. (C) Cell cycle phase estimation of single undifferentiated germ cells from wildtype, *nos-Gal4::VP16>UASz-EcR-B1*, *nos-Gal4::VP16>UASp-tkv*^{ACT}, and *bam*$^{\Delta 86}$ ovaries, based on CellCycleScoring (Zhu et al., 2022) of cell cycle transcripts in single cell mRNA datasets. Numbers at the bottom of each column represent undifferentiated germ cell subtype (0-5).

Development • Supplementary information

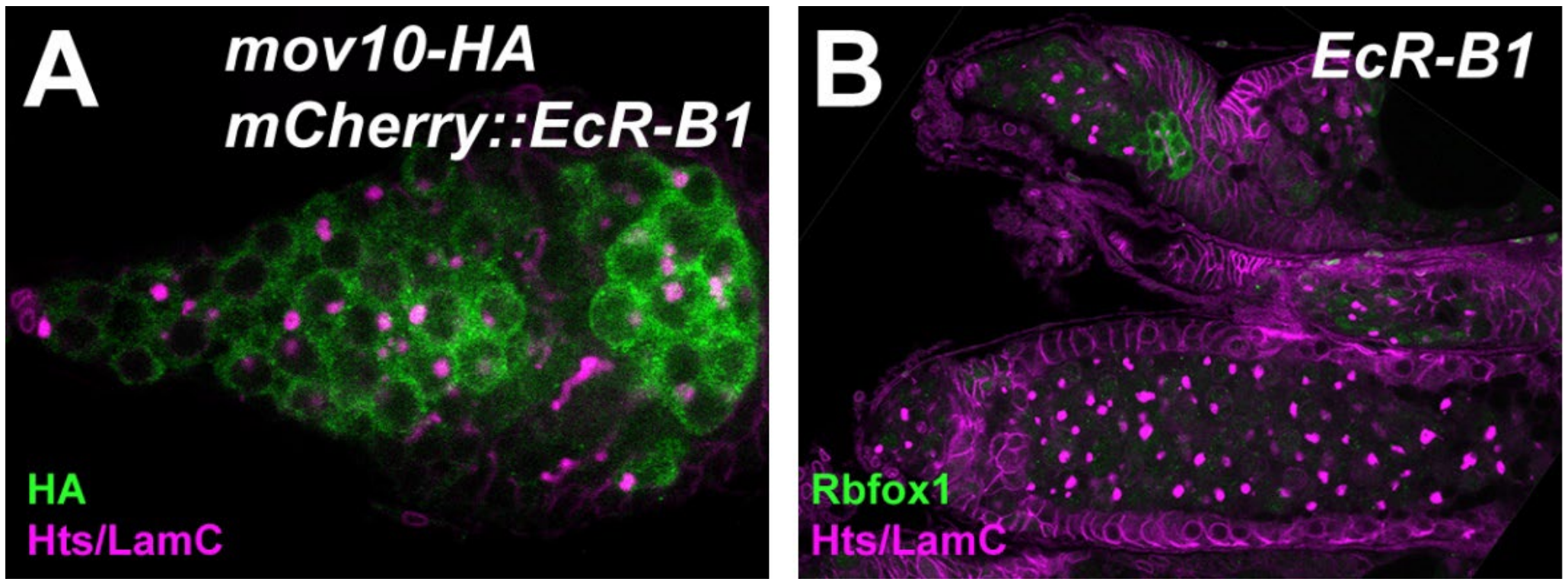

Fig. S8. Over-expression of *EcR-B1* in undifferentiated cells delays differentiation prior to down-regulation of *Mov10* and up-regulation of *Rbfox1*. *nos-Gal4::VP16>UASz-EcR-B1, mov10$^{KI\text{-}HA}$* ovaries immunostained for HA (A; *Mov10* reporter; green) or Rbfox1 (B; green), Hts (magenta; fusomes and follicle cells), and LamC (magenta; cap cells and stalk cells).

Development • Supplementary information

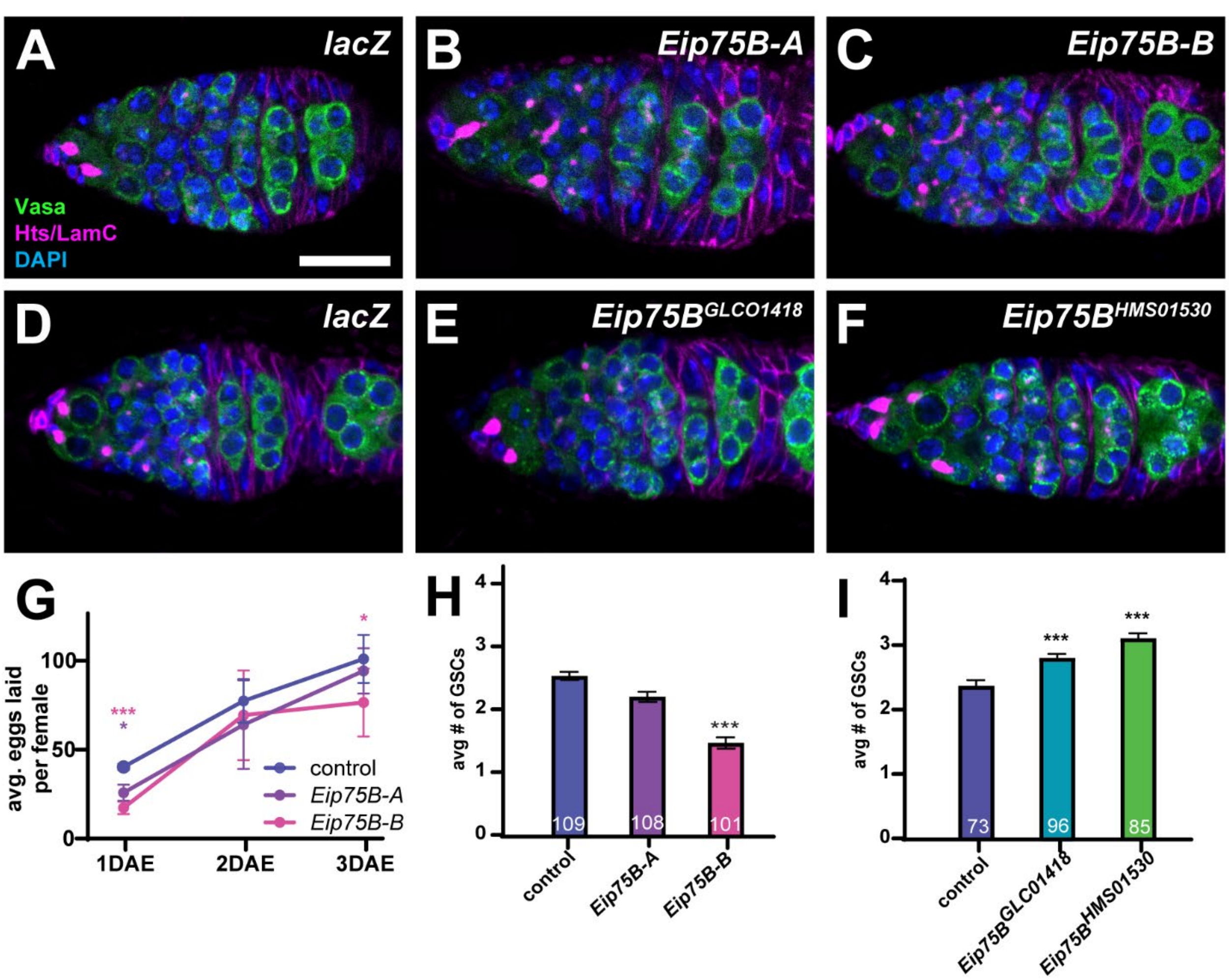

Fig. S9. *Eip75B* is necessary and sufficient to repress GSC self-renewal, but is dispensible for germ cell differentiation. (A-F) Representative images (A-F) and quantification (G-I) of control (A,D), *nos-Gal4::VP16>UASz-Eip75B* over-expression (B-C), or depletion of *Eip75B* via germline-enhanced RNAi (*Eip75B*^{GLC01418} and *Eip75B*^{HMS01530}) germaria immunostained for Vasa (germ cells; green), Hts (magenta; fusomes and follicle cells), LamC (magenta; cap cells and stalk cells), and DAPI (nuclei). Scale bars = 10 µm. (G) Average number of eggs laid per female over the first three days after eclosion (DAE). (H-I) Average number of GSCs per germarium at 5 days after eclosion. Numbers in bars represent number of germaria scored. *p<0.01, ***p<0.001, compared to control; Student's t-test.

Development • Supplementary information

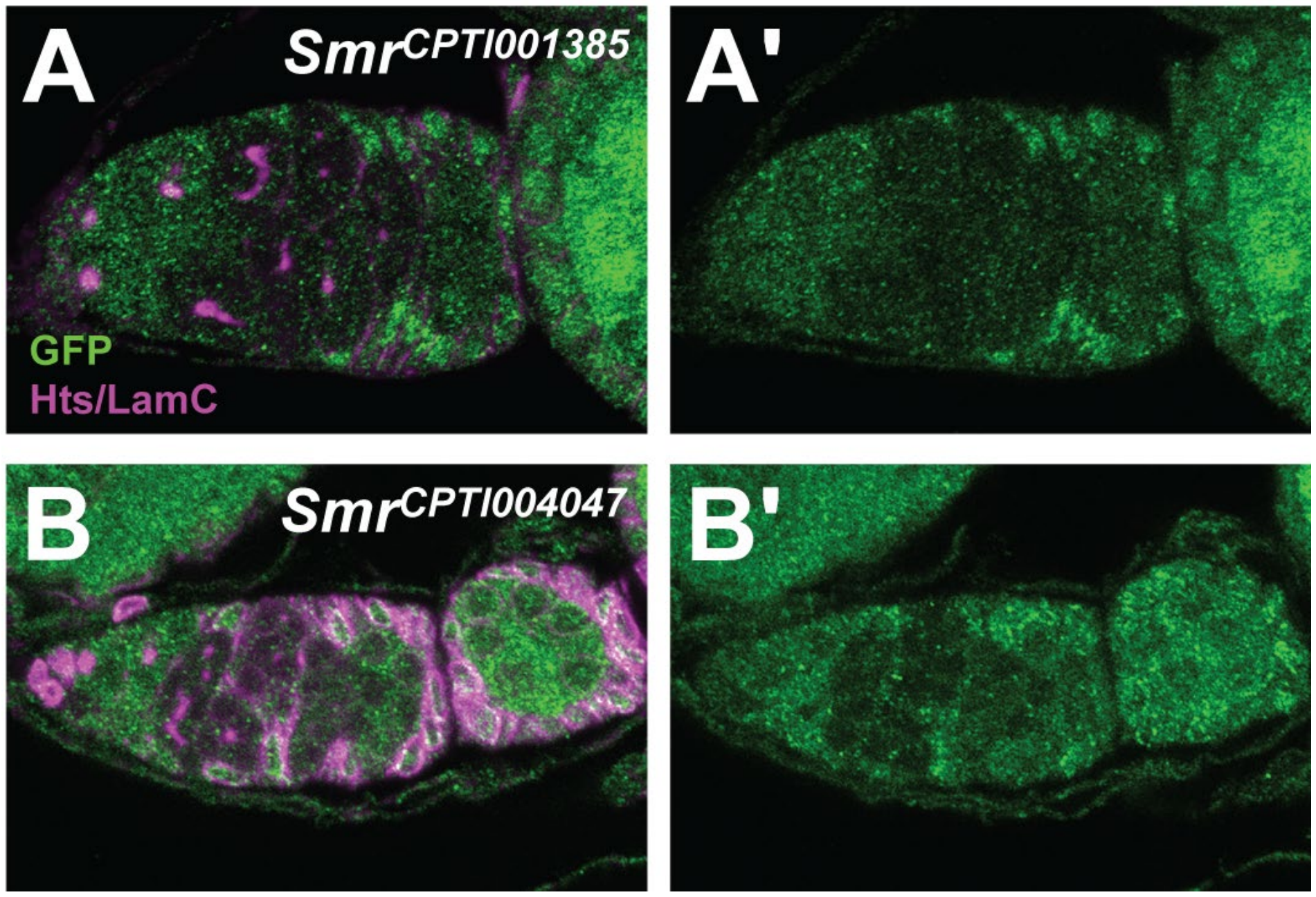

Fig. S10. The co-repressor *Smr* is expressed in undifferentiated germ cells. Flies carrying GFP-tagged protein trap *Smr* reporters (A-A', $Smr^{CPTI001385}$; B-B', $Smr^{CPTI004047}$) immunostained for GFP (reporter expression; green), Hts (magenta; fusomes and follicle cells), and LamC (magenta; cap cells and stalk cells).

Development • Supplementary information

Table S1. Key resources used in this study.

***Drosophila* Stocks**		
**Stock Name**	**Source**	**Identifier**
UASp-lacZ	D. Drummond-Barbosa	Bloomington *Drosophila* Stock Center (BDSC) #98113
w^{1118}; P[GAL4::VP16-nanos.UTR]$CG6325^{MVD1}$ (nos-Gal4::VP16)	D. Drummond-Barbosa (Rørth, 1998; Van Doren et al., 1998)	BDSC #4937 or #7253
w^{*}; P[Act5C-GAL4]25FO1/CyO; M[3xP3-GFP.R]ZH-86Fb	BDSC	BDSC #78574
y^{1} w^{*}; TI[CRIMIC.TG4.1]$aub^{CR02423\text{-}TG4.1}$/SM6a (Aub-Gal4)	BDSC	BDSC #92663
P[otu-GAL4::VP16.R]1, w^{1118}	BDSC	BDSC #58424
bam>Gal4^A,Bam>Gal4^C/CyO;Bam>Gal4/TM6 (3xbam-Gal4)	J. Mathieu & J.-R. Huynh (Clémot et al., 2018)	
UASz-EcR-$B1^{DC655}$ (attP40)/CyO	this manuscript	
UASz-mCherry::EcR-$B1^{DC655}$ (attP40)/CyO	this manuscript	
UASz-mCherry::EcR-$B1^{DC655}$ (attP2)/TM6	this manuscript	
UAS-$EcR^{RNAi1\ (Valium22\text{-}attP2)}$/TM3	this manuscript	
UAS-$EcR^{RNAi2\ (Valium22\text{-}attP2)}$/TM3	this manuscript	
UAS-$EcR^{RNAi3\ (Valium22\text{-}attP2)}$/TM3	this manuscript	
UASz-HA::Eip75B-A (attP2) / TM3	this manuscript	
UASz-HA::Eip75B-B (attP2) / TM3	this manuscript	
UASp-tkv^{Q199D}/TM6	D. Drummond-Barbosa (Bolívar et al., 2006)	
ry^{506} e^{1} $bam^{\Delta 86}$/TM3, ry^{RK} Sb^{1} Ser^{1}	BDSC	BDSC #5427
y^{1} v^{1}; P[TRiP.HMJ22155]attP40 (bam RNAi)	BDSC	BDSC #58178
y^{1} v^{1}; P[TRiP.HMS02185]attP40 (tkv RNAi)	BDSC	BDSC #40937
y^{1} sc^{*} v^{1} sev^{21}; P[TRiP.HMS04501]attP40 (tkv RNAi)	BDSC	BDSC #57303
dpp^{hr56} cn^{1} bw^{1}/CyO	BDSC	BDSC #36528
dpp^{e87} cn^{1} bw^{1}/CyO	BDSC	BDSC #2073
PBac[fTRG00003.sfGFP-TVPTBF]VK00033 (Bam::GFP)	Vienna Drosophila Resource Center	v318001
Dad-lacZ (likely P[lacZ]Dad^{P1883})	D. Drummond-Barbosa (Kai and Spradling, 2003; Song et al., 2004)	
y^{1} w^{*}; Mi[PT-GFSTF.1]Eip63EMI00413-GFSTF.1/TM6C, Sb^{1} Tb^{1}	BDSC	BDSC #59763
w; dMov10[KI, smGFP-HA]/SM; Pr.e/TM3 Ser	H. Nakato (Takemura et al., 2021)	
y^{1} w^{*}; Mi[MIC]$Eip93F^{MI05200}$/TM3, Sb^{1} Ser^{1}	BDSC	BDSC #43675

y^1 w^*; *P[E(bx)-GFP.FPTB]attP40*	BDSC	BDSC #68180
y^1 w^*; *PBac[crol-GFP.FPTB]VK00031*	BDSC	BDSC #93087
w^{1118}; *PBac[HmgD-GFP.FPTB]VK00033*	BDSC	BDSC #55827
w^{1118}; *PBac[Hr78-GFP.FLAG]VK00037*	BDSC	BDSC #38653
w^{1118}; *PBac[Hnf4-GFP.FLAG]VK00033*	BDSC	BDSC #38649
y^1 w^*; *PBac[Dp-GFP.FPTB]VK00033*	BDSC	BDSC #67388
w; P[UASp-hts.mCherry]attP2*	BDSC	BDSC #66171
w^{1118}; *PBac[lola.DE-GFP.FLAG]VK00033/TM3, Sb*^{1}	BDSC	BDSC #43948
w^{1118}; *PBac[lola.I-GFP.FLAG]VK00033*	BDSC	BDSC #38662
y^1 w^{67c23}; *ftz-f1*^{ex7} *P[FRT(w*^{hs}*)]2A/TM6B, P{Ubi-GFP.S65T}PAD2, Tb*$^+$	BDSC	BDSC #64338
w^*; *P[FRT(w*^{hs}*)]2A (FRT79D)*	BDSC	BDSC #1997
hsFLP; nGFP FRT79D/TM3	M. Buszczak	
w^{1118} *PBac[806.LOX-SVS-2]Smr*^{CPTI004047}	Kyoto *Drosophila* Stock Center	115465
w^{1118} *PBac[681.P.FSVS-1]Smr*^{CPTI001385}	Kyoto *Drosophila* Stock Center	115513
**Antibodies and Stains**		
**Antibody**	**Source**	**Identifier**
Mouse anti-Hts (1:10)	Developmental Studies Hybridoma Bank (DSHB)	1B1; AB_528070
Mouse anti-LamC (1:100)	DSHB	LC28.26; AB_528339
Chicken anti-GFP (1:2000)	Abcam	AB_13970
Rabbit anti-Vasa (1:1000)	P. Lasko (Lasko and Ashburner, 1990)	
Rabbit anti-Vasa (1:1000)	Boster Biological	DZ41154
Chicken anti-Vasa (1:5000)	P. Rangan (Upadhyay et al., 2016)	
Chicken anti-b gal (1:2000)	Abcam	AB_9361
Rabbit anti-DsRed (1:500)	Takara	632496
Rabbit anti-Tkv2 (Tkv-A) (1:250)	M. O'Connor (Peterson et al., 2022)	
Guinea pig anti-Rbfox1 (1:5000)	M. Buszczak (Tastan et al., 2010)	
Rabbit anti-pMad (1:100)	Abcam	AB_52903
Mouse anti-Orb (1:500)	DSHB	4H8; AB_528418
Mouse anti-Orb (1:500)	DSHB	6H4; AB_528419
Rabbit anti-Blanks (1:1000)	Erik Sontheimer (Gerbasi et al., 2011)	
Rat anti-Chinmo (1:1000)	Nick Sokol and Lesley Weaver (Wu et al., 2012)	
Mouse anti-Lola (1:20)	DSHB	7F1-1D5 RRID: AB_2721954

Development • Supplementary information

Rabbit anti-pHistone H3 (pHH3) (1:200)	Millipore	06-570
Click-iT EdU Cell Proliferation Kit for Imaging, Alexa Fluor 647 dye	Life Technologies	C10340
Goat anti-mouse AlexaFluor 488, 568, 633 (1:200)	Life Technologies	
Goat anti-chicken AlexaFluor 488 (1:200)	Life Technologies	
Goat anti-rabbit AlexaFluor 488, 568 (1:200)	Life Technologies	
Goat anti-rat Alexa Fluor 488 (1:200)	Life Technologies	
4'-6-diamidino-2-phenylindole (DAPI) (0.5 mg/mL)	Sigma	

**Primers**	
**Primer Name**	**Sequence (5'-3')**
UASp-Tkv^{ACT} forward	GTCATCAAGCTTAGGCCTCCAA
UASp-Tkv^{ACT} reverse	TCCCGGTCGTCTCATCGTAA
pVALIUM 22 forward	GGT GAT AGA GCC TGA ACC AG
pVALIUM 22 reverse	TAA TCG TGT GTG ATG CCT ACC
UASz-EcR.B1 C Term forward	GCTGTACAAGATGAAGCGGCGCTGGTCG
UASz-EcR.B1 C Term reverse	AGTGGTACCCTCGAGGGATCCTAGATGGCA TGAACGTCCCAG
EcR common forward (exon 3-4)	CCTCCGGCTACCACTACAAC
EcR common reverse	TATTGCGCGCTTGACACTTG
EcR-A forward (exon 2-3)	CGCCGGAAGCTATGATCCTT
EcR-A reverse	TCCTTCTCCTTCTGGGCCTT
EcR-B forward (exon 1-3)	ATGGCCTAAGTCAGCAGCAG
EcR-B forward	CTTGCTCTTCTTCGCATCGC

Development • Supplementary information

Table S2. Combined single cell mRNA expression from four genotypes (wild-type, *EcR-B1*, *tkv*^{ACT}, and *bam*$^{\Delta 86}$), per cell cluster. Gene expression data used to assign cell type identities to each cluster. Relates to Figures 3-5 and 6A.

Available for download at
https://journals.biologists.com/dev/article-lookup/doi/10.1242/dev.205460#supplementary-data

Table S3. Combined single cell mRNA expression from four genotypes (wild-type, *EcR-B1*, *tkv*^{ACT}, and *bam*$^{\Delta 86}$), including undifferentiated germ cells only. Gene expression data used to assign cell type identities to each cluster. Relates to Figures 3-5 and 6A.

Available for download at
https://journals.biologists.com/dev/article-lookup/doi/10.1242/dev.205460#supplementary-data

Table S4. Results from SCENIC analyses, predicting transcription factors regulating gene expression in each undifferentiated cell cluster. Relates to Figures 6H and 7A.

Available for download at
https://journals.biologists.com/dev/article-lookup/doi/10.1242/dev.205460#supplementary-data

Supplementary references

Bolívar, J., Pearson, J., López-Onieva, L. and González-Reyes, A. (2006). Genetic dissection of a stem cell niche: the case of the Drosophila ovary. *Dev Dyn* **235**, 2969–2979.

Clémot, M., Molla-Herman, A., Mathieu, J., Huynh, J. R. and Dostatni, N. (2018). The replicative histone chaperone CAF1 is essential for the maintenance of identity and genome integrity in adult stem cells. *Development* **145**.

Gerbasi, V. R., Preall, J. B., Golden, D. E., Powell, D. W., Cummins, T. D. and Sontheimer, E. J. (2011). Blanks, a nuclear siRNA/dsRNA-binding complex component, is required for Drosophila spermiogenesis. *Proc Natl Acad Sci U S A* **108**, 3204–3209.

Kai, T. and Spradling, A. (2003). An empty Drosophila stem cell niche reactivates the proliferation of ectopic cells. *Proc Natl Acad Sci U S A* **100**, 4633–4638.

Karim, F. D. and Thummel, C. S. (1992). Temporal coordination of regulatory gene expression by the steroid hormone ecdysone. *Embo j* **11**, 4083–4093.

Lasko, P. F. and Ashburner, M. (1990). Posterior localization of vasa protein correlates with, but is not sufficient for, pole cell development. *Genes Dev* **4**, 905–921.

Peterson, A. J., Murphy, S. J., Mundt, M. G., Shimell, M., Leof, E. B. and O'Connor, M. B. (2022). A juxtamembrane basolateral targeting motif regulates signaling through a TGF-β pathway receptor in Drosophila. *PLoS Biol* **20**, e3001660.

Romani, P., Gargiulo, G. and Cavaliere, V. (2016). The ecdysone receptor signalling regulates microvilli formation in follicular epithelial cells. *Cell Mol Life Sci* **73**, 409–425.

Rørth, P. (1998). Gal4 in the Drosophila female germline. *Mech Dev* **78**, 113–118.

Song, X., Wong, M. D., Kawase, E., Xi, R., Ding, B. C., McCarthy, J. J. and Xie, T. (2004). Bmp signals from niche cells directly repress transcription of a differentiation-promoting gene, bag of marbles, in germline stem cells in the Drosophila ovary. *Development* **131**, 1353–1364.

Takemura, M., Bowden, N., Lu, Y. S., Nakato, E., O'Connor, M. B. and Nakato, H. (2021). Drosophila MOV10 regulates the termination of midgut regeneration. *Genetics* **218**.

Tastan, O. Y., Maines, J. Z., Li, Y., McKearin, D. M. and Buszczak, M. (2010). Drosophila ataxin 2-binding protein 1 marks an intermediate step in the molecular differentiation of female germline cysts. *Development* **137**, 3167–3176.

Upadhyay, M., Martino Cortez, Y., Wong-Deyrup, S., Tavares, L., Schowalter, S., Flora, P., Hill, C., Nasrallah, M. A., Chittur, S. and Rangan, P. (2016). Transposon Dysregulation Modulates dWnt4 Signaling to Control Germline Stem Cell Differentiation in Drosophila. *PLoS Genet* **12**, e1005918.

Van Doren, M., Williamson, A. L. and Lehmann, R. (1998). Regulation of zygotic gene expression in Drosophila primordial germ cells. *Current biology : CB* **8**, 243–246.

Wu, Y. C., Chen, C. H., Mercer, A. and Sokol, N. S. (2012). Let-7-complex microRNAs regulate the temporal identity of Drosophila mushroom body neurons via chinmo. *Developmental cell* **23**, 202–209.
