# Supplementary material for: Ecdysone receptor autonomously controls germ cell differentiation in the Drosophila ovary
Source: Development. 2026 Jul 29;153(14):dev205460. doi: 10.1242/dev.205460 (PMC13489555; doi:10.1242/dev.205460)
Supplement: Supplementary file 2 [file develop-153-205460_review_history.pdf]

Development

Ecdysone receptor autonomously controls germ cell differentiation in the *Drosophila* ovary

Lauren E. Jung, Alexandria I. Warren, Changhong Yin, Weihua Huang, Allison C. Simmons, Samantha I. McDonald, Lindsay A. Swain, Victoria E. Garrido, Daniel N. Phipps, BiClaireline Cesar, Danielle S. Finger, Zhipeng Sun, Todd G. Nystul and Elizabeth T. Ables
DOI: 10.1242/dev.205460

Editor: Cassandra Extavour

Review timeline

Original submission:	17 December 2025
Editorial decision:	2 February 2026
First revision received:	6 May 2026
Accepted:	18 June 2026

Original submission

First decision letter

MS ID#: dev.205460

MS TITLE: Ecdysone Receptor autonomously controls germ cell differentiation in the Drosophila ovary

AUTHORS: Lauren E. Jung; Alexandria I. Warren; Changhong Yin; Weihua Huang; Allison C. Simmons; Samantha McDonald; Lindsay A. Swain; Victoria E. Garrido; Daniel N. Phipps; BiClaireline Cesar; Danielle S. Finger; Zhipeng Sun; Todd G. Nystul; Elizabeth T. Ables

Dear Dr Ables,

I have now received all the referees reports on the above manuscript, and have reached a decision. The referees' comments are appended below.

The overall evaluation is positive and we would like to publish a revised manuscript in Development, provided that the referees' comments can be satisfactorily addressed. Please attend to all of the reviewers' comments in your revised manuscript and detail them in your point-by-point response. If you do not agree with any of their criticisms or suggestions explain clearly why this is so. If it would be helpful, you are welcome to contact us to discuss your revision in greater detail. Please send us a point-by-point response indicating your plans for addressing the referees' comments, and we will look over this and provide further guidance.

Reviewer 1

SUMMARY OF THE ADVANCE MADE IN THIS PAPER AND ITS POTENTIAL SIGNIFICANCE TO THE FIELD

The study by Jung et al. investigated the autonomous role of Ecdysone receptor (EcR) in germline development in Drosophila ovaries. First, they evaluated the expression of EcR in germline stem cells (GSCs) and early germ cells. Using germline-specific EcRRNAi or EcRDN, they went on to show that EcR knockdown in germlines mildly reduces GSC self-renewal. Next, they demonstrate that

© 2026. Published by The Company of Biologists under the terms of the Creative Commons Attribution License (https://creativecommons.org/licenses/by/4.0/).

Overexpression of EcR, as well as some of EcRDN, leads to germline tumor phenotype, which phenocopies bam mutants and constitutive activation of BMP signaling. Subsequently, these authors performed scRNA-seq analysis and characterized germline similarity between EcR overexpression, tkvAct, and bam mutant. Ultimately, the authors conclude that EcR functions autonomously controls germ cell differentiation in the Drosophila ovary.

Ecdysone signaling has been shown to regulate GSC in an non-cell autonomous manner previously. The autonomous role of ecdysone signaling or EcR in GSC and germline differentiation hasn't been demonstrated in the field. Therefore, the current work is novel and some of the phenotype is striking and interesting. However, in multiple cases, experimental data are conflicting, and conclusions are not substantially supported by the experimental data. The gain-of-function data need to be cautiously interpreted and may not be appropriate for inferring the physiological role of EcR. The rational for some of the experiments are not clearly stated. The manuscript need to be substantially modified before publications. Here are some of the major and minor concerns listed below as suggestions to authors.

SUGGESTIONS TO AUTHORS

Major concerns:

1. Line 147 describes the EcR expression in GSCs (Figure 1B). It is unclear whether the antibody staining in GSCs is real signal for EcR protein and needs validation. An easy experiment is to compare this to nos>EcRRNAi germarium to see whether EcR staining is indeed knocked down.

2. Line 152-153 described "EcRRNAi1, which targets all EcR isoforms; Figure S1A-C". This is confusing and inconsistent with Figure S1A showing that EcRRNAi1 targets EcR.A isoform. In addition, Figure S1C showed that EcR.B1 isoform level is also affected by EcRRNAi1. It is very confusing why EcRRNAi1 can affect EcR.B1 level if it targets EcR.A isoform specifically according to the hairpin structure. In addition, EcRRNAi3 showed no detectable EcR mRNA level in the entire ovaries, which is inconsistent with the fact that somatic follicle cells should have EcR mRNA expression. This leads the reviewer to question the validity of this method to validate the RNAi efficacy using the ovaries, since the cell composition in the ovaries are potentially different in different genotypes.

3. Figure 1 showed EcRRNAi1 affects the GSC number; however, it is confusing why FLP clone system (Figure 1J) is stronger than the constitutive Gal4 knockdown (Figure 1G). In addition, EcRRNAi2 and EcRRNAi3 seemed more efficient than EcRRNAi1 (Figure S1), it is necessary to use these RNAi lines to recapitulate the phenotype, particularly considering that EcRDN does not show the GSC loss phenotype.

4. There's no explanation why EcRDN lines do not show GSC loss in Line 160-162. If results are indeed repeatable, it is likely suggesting that there's no ecdysone signaling/activity in GSC and that EcRRNAi phenotype could be due to that EcR/USP functions as a transcriptional repressor in GSC without ecdysone binding. A way to differentiate these scenario is to use ecdysone ligand sensor to determine whether ecdysone ligands can be detected in GSCs.

5. The conclusion that "Over-expression of EcR in GSCs and cystoblasts delays germ cell differentiation in a ligand-dependent manner" in Line 168-169 is not supported by the experimental data. The experimental data is rather confusion and conflicting in Figure 2.
I. Authors showed that EcR-B1W650A blocked germline differentiation, while EcR-B1F645A and EcR-B1DBL did not in Line196-199. Authors postulate that EcR-B1F645A/EcR-AF617A functions as ligand sponges to affect endogenous ecdysone level. If that is the case, EcR-B1DBL cannot bind ligand and should show the same phenotype as EcR-B1W650A, which is completely opposite (Figure 1J).
II. In addition, authors used the ecdysone feeding to partially rescue EcR-B1 phenotype to support their claim; however, it will be important to show that ecdysone feeding cannot rescue EcR-B1W650A phenotype as EcR-B1W650A cannot bind to ecdysone.

6. There's no detailed description of the re-clustering of undifferentiated germ cells. It is unclear the accuracy of the subsequent analysis. It seems that each clusters in different genetic

© 2026. Published by The Company of Biologists under the terms of the Creative Commons Attribution License (https://creativecommons.org/licenses/by/4.0/).

backgrounds are very different. It will probably better to combine all the undifferentiated germ cells from all genotypes for re-clustering to find similar cells.

7. Line 259-260 described that tkv "was up-regulated in response to both EcR-B1 and tkvAct over-expression (Figure 4H)". It is not so obvious that tkv was upregulated in both EcR-B1 and tkvAct overexpression in comparison to bamΔ86 mutant. In the subsequent Tkv antibody analysis (Figure 4K-M), it will be better to include bamΔ86 mutant to confirm this conclusion and support that EcR-B1 overexpression indeed upregulates Tkv expression.

8. In line 430-432, it is unclear why authors tested Lola-DE isoform. In addition, the data are conflicting to each other. Why did anti-Lola-DE staining show different expression pattern as lola-DE reporter line? How can these data support the conclusion that Lola influence gene expression programs during germ cell differentiation.

Minor concerns:

1. Line 147-149 concludes that EcR levels were lower in undifferentiated germ cells than in 16-cell cysts and lower in the germline compared to somatic follicle cells. It is not obvious from the images shown in Figure 1B-C. Quantification is required to substantiate this statement.

2. It will be better to replace EcRRNAi to EcRRNAi1 in Figure 1F-G and Figure 1I-J to increase the clarity.

3. Line 231-232 indicates HP6 and minidiscs to differentiate undifferentiated and differentiated germlines, respectively (Figure S3). Figure S3 did not show minidiscs expression. Please add the expression panel to confirm the clustering.

4. In Figure 5, the cluster-enriched genes are not obviously show in specific clusters. An outline of each cluster may help for better visualization.

5. There's no supplemental dataset to support the analysis in Figure 6A, as well as Figure 6H and 7A.

Reviewer 2

SUMMARY OF THE ADVANCE MADE IN THIS PAPER AND ITS POTENTIAL SIGNIFICANCE TO THE FIELD

Using genetic tools, the authors provided strong evidence the balanced ecdysone receptor (EcR) activity functions within Drosophila ovarian germline stem cells to control the balance between stem cell renewal and differentiation. In addition, they uncover transcriptional targets, identified by mining previously published scRNAseq datasets of the ovary, of EcR that mediate its GSC-autonomous function. The group also uses the scRNAseq data to parse out distinct features of GSC-like cells elicited by different genetic manipulations, thus uncovering different mechanisms by which these cell types accumulate. Altogether, this works advances understanding of how a single hormone mediates different cellular responses in various cell types.

SUGGESTIONS TO AUTHORS

Moderate concerns

1. Lines 147-149 and Figure 1B,C: In general, different EcR expression levels are evident in the representative images, but this could be supported better by quantification. Additionally, there is a discrepancy when comparing somatic versus germline EcR. The text states "…lower in the germline compared to somatic follicle cells until stage 10…", but the staining in the germline seems to be higher than in follicle cells in the region 3 and stage 1 egg chamber shown in Fig 1

2. Lines 299-322 and Figure 5: Assessing expression patterns of the genes shown in the mutant backgrounds would further confirm the transcriptomics data. I do understand that doing so for all reporter-background combinations might be unreasonable, but a subset might be feasible.

Minor concerns

© 2026. Published by The Company of Biologists under the terms of the Creative Commons Attribution License (https://creativecommons.org/licenses/by/4.0/).

1. Figure 1F: The anterior-most tip of the germarium shown appears to be tucked under, thus not including the niche.
2. Figure 2: The order of manipulations shown for the representative images is not the same as the order shown for the quantification
3. Related to "Moderate concern" 2: The data describe in lines 339-344 and shown in Figure S7 addresses the moderate concern 2, and may fit better with that section
4. Figure 7F: The colors referred to in the figure legend do not match with the actual model schematic. Additionally, there is some disconnect between what is described and the "signal bars" in the stem versus egg cell. Specifically, the bars in the stem cell should be filled in for the "stem cell" genes and empty for the "egg" and the reverse should be true for the egg cell. Instead, this is what is shown for the egg cell.

<u>Reviewer 3</u>

In the manuscript "Ecdysone receptor autonomously controls germ cell differentiation in the Drosophila Ovary" The authors describe experiments examining the developmental impact of manipulating ecdysone signaling in the germline stem cells (GSCs) of the Drosophila ovary. Prior work had described non-cell autonomous roles for ecdysone signaling in regulating stem cell differentiation through supporting somatic cells. However, this study provides convincing data demonstrating that ecdysone signaling functions cell autonomously within the GSCs to regulate both GSC maintenance and differentiation. Through additional experiments that expand on this initial observation, the authors additionally demonstrate that ecdysone likely influences GSC maintenance and differentiation through different autonomous mechanisms. The authors also provide experiments that demonstrate how ecdysone signaling interacts with other known pathways that regulate germline differentiation in the ovary, providing evidence that ecdysone signaling collaborates with BMP signaling to regulate germline differentiation, while also demonstrating with scRNA data that the qualitatively similar phenotypes produced by these different manipulations (excess undifferentiated germ cells) reflect distinct populations of GSC-like progenitor cells. Finally, through analysis of single cell sequencing data the authors identify and confirm multiple transcriptional targets that may mediate ecdysone/BMP signaling during differentiation.

While this manuscript focuses on experiments and mechanisms in the Drosophila ovary, I the findings here addresses several issues that would be of general interest to the broad readership of this journal, including 1) a demonstration that endocrine signals can cell-autonomously direct stem cell maintenance and differentiation, 2) a model for how endocrine signals can produce discrete responses within a tissue and even within cell lineages through modulation of hormone receptor titer and interactions with other signaling pathways. 3) An example of how developmental pathway manipulation can produce phenotypically similar, but transcriptionally distinct developmental delays/arrests.

The experiments in this manuscript appear to be well executed and there is plenty of data that will be valuable to those researchers who are specifically studying this model, which should allow them to build on these findings. However, the questions/suggestions I have below mainly address the interpretation of the different EcR alleles and suggestions on ways the authors could make the manuscript more impactful by making it clearer and more accessible to a broader audience.

Major comments:
1) Interpretation of EcR allele results
I found it difficult to fully interpret the different results that were observed with the different EcR alleles in both GSC maintenance and germ cell differentiation. In some cases, it would be beneficial for the authors to explain their interpretation of the differences they observe. In other cases, I found the explanations confusing and could either use clarification or reinterpretation. For instance:

A. For the GSC maintenance experiments in Figure 1, how do the authors interpret the differences between the EcR RNAi effect on GSC number vs. the failure of either the activation mutants (F645A) or double mutants with the ligand binding mutant (DBL) to produce a similar phenotype? It's unclear to me how to interpret these differences. Was the W650A allele examined on its own in this assay?

© 2026. Published by The Company of Biologists under the terms of the Creative Commons Attribution License (https://creativecommons.org/licenses/by/4.0/).

B. I found the description of the experiments to examine EcR allele effect on germ cell differentiation and subsequent interpretation in lines 193-203 to be confusing. I believe that because OX of EcR-B1 along with the binding defective allele (W650A) both limit differentiation, whereas F645A (activation defective) and DBL (both binding and activation defective) don't limit differentiation led the authors to: "…postulate that they [unclear what "they" refers to… EcR-B1OX and W650A?] function as ligand sponges, effectively shunting endogenous EcR activity towards transcriptional repression." But it's unclear to me how the binding defective allele acts as a ligand sponge and why the F645 allele, which still retains binding, and so could act as a ligand sponge, doesn't produce a phenotype. Is the phenotype resulting from EcR-B1 overexpression due to increased expression of endogenous EcR target genes? Or increased suppression of endogenous EcR target genes through the additional EcR-B1 sequestering ligand from endogenous sites? While rescue of EcR-B1 expressing larvae with endogenous ecdysone might support the sponge hypothesis, would you expect ecdysone feeding to rescue the W650A allele, which can't bind ecdysone? It seems like there are some inconsistencies that belie a simple explanation here and it would be helpful to have a clearer sense of how we can interpret the EcR alleles, especially EcR-B1 overexpression.

C. This issue carries over into the subsequent expression analysis to identify "ecdysone responsive genes" through the comparison of EcR-B1OX vs tkv[ACT], and bam[delta86]. especially in thinking about whether the gene expression changes observed reflect a direct effect of EcR activation of the target vs. indirect activity of EcR-B1 OX leading to endogenous EcR producing transcriptional repression. It also comes up in the summary Figure 7F… does EcR/USP activate Tkv? Or is the increase of Tkv in the EcR-B1 Ox an indirect effect of increased EcR repression at endogenous sites? A better clarification and explanation of how these alleles are acting would make these results easier to interpret and clarify the model for a general audience.

D. While EcR-B1 overexpression produces a qualitatively bam phenotype independently of tkv or dpp, is the converse true? Does the bam or tkv[ACT] phenotype depend on EcR signaling? Is it suppressed by EcR DN or RNAi? Does the result described in lines 382-384, Figure 6H suggest that the bam mutant phenotype might depend on EcR activity?

Minor suggestions:

1) Line 152 says that EcR RNAi1 targets all EcR isoforms. However, in figure S1 it appears that the EcR RNAi1 would be targeted to the EcR A isoform and the RNAi2 and RNAi3 target the common exons. Am I misinterpreting this figure?

2) For clarity, I think the mCherry-tagged EcR-A and B1 alleles in Figure 2 could be moved into supplemental data or omitted, I don't think the analysis of those alleles is necessary for the overall conclusions.

3) Line 114, suggest "received by GSCs via"

4) Line 149, should "until" be "at"? after stage 10 is germline expression equal/higher than follicle cells?

5) For accessibility, it would be helpful to label the components of the stage 10 image in Figure 1C for a general audience.

6) In Figure 1 E-H it would be good to label cells with arrows/arrowheads since the green and purple label multiple cell types.

7) In Figure S4, it would be helpful to a general audience if the authors could better annotate the phenotype that is being evaluated in these images.

In summary, I think this manuscript makes a valuable contribution to our understanding of GSC differentiation in the Drosophila ovary and provides a model for understanding hormonal regulation in complex tissues that would be appreciated by the broader readership of Development. I believe better clarification of the interpretation of the different EcR alleles and an effort throughout to

© 2026. Published by The Company of Biologists under the terms of the Creative Commons Attribution License (https://creativecommons.org/licenses/by/4.0/).

better communicate the results to a broader audience less familiar with Drosophila nomenclature and ovary anatomy would make this manuscript quite suitable for publication in Development.

First revision

Author response to reviewers' comments

DEVELOP-S-25-00994 – Response to Reviewers

We would first like to thank the reviewers for providing constructive comments and suggestions to strengthen our manuscript, and to the editor for their positive assessment of our work. We are very excited that the overall evaluation is positive, and that the editor indicated their desire to publish our revised manuscript.

In general, the reviewers were very pleased with the manuscript, acknowledging the novelty and potential impact of the study on the field. They noted that this work "advances understanding of how a single hormone mediates different cellular responses in various cell types" (Reviewer 2) and "addresses several issues that would be of general interest to the broad readership of this journal" (Reviewer 3). Notably, Reviewer 3 explained that "this manuscript makes a valuable contribution to our understanding of GSC differentiation in the *Drosophila* ovary and provides a model for understanding hormonal regulation in complex tissues that would be appreciated by the broader readership of *Development*."

The reviewers also noted areas for improvement in the manuscript, which we summarize here:

1. **Interpretation and consistency of the EcR manipulation data.** Across all Reviewers, the largest concern is that the effects of different EcR alleles and perturbations (RNAi, over-expression, ligand-binding mutants) are internally inconsistent and insufficiently explained.
   - EcR RNAi causes GSC loss, but the EcR^{F645A} dominant-negative allele does not, calling into question whether EcR signaling is active in GSCs or whether EcR functions as a ligand-independent repressor.
   - Different EcR mutant alleles (EcR^{W650A}, EcR^{F645A}, EcR^{DBL}) produce unexpectedly divergent phenotypes that are not convincingly reconciled with their molecular functions.
   - Over-expression phenotypes (tumor-like germline accumulation) may be non-physiological artifacts.
   - Reviewers requested a clearer mechanistic interpretation of how EcR activity (activation vs repression, ligand-dependent vs independent) leads to the observed phenotypes.

Like the Reviewers, we are also intrigued by the apparent inconsistency between the EcR manipulations! We believe that some of the issues raised by the reviewers can be addressed by including a deeper analysis of the EcR RNAi data (see response to Reviewer 1, below). To summarize, we performed fertility assays (egg counts), GSC assays (number of GSCs per germarium, in a static timepoint and in clonal analyses), and semi-quantitative RT-PCR to validate the RNAi transgenes. In each assay, we observed statistically significant reductions in each of these metrics in RNAi lines as compared to age-matched wildtype controls (data below). We initially only included results from GSC assays for one EcR RNAi line, as this made presentation of the data clearer; however, repetition of the experiment in two other RNAi lines yielded similar results, bolstering our conclusion that EcR is endogenously necessary in the ovarian germline to sustain GSC number. In the revised manuscript, we include that data in a new supplemental figure (Figure S1). The fact that the EcR RNAi GSC phenotype is quite mild compared to other regulators of GSC self-renewal likely means that EcR is more important to block differentiation than to maintain self-renewal, and we clarified this point in the text.

This leads to the question of why expression of EcR RNAi and EcR dominant negative lines do not yield the same GSC phenotypes. It is important that we note here that the EcR^{F645A},

© 2026. Published by The Company of Biologists under the terms of the Creative Commons Attribution License (https://creativecommons.org/licenses/by/4.0/).

EcR^{DBL}, and EcR RNAi transgenes share an encapsulation delay phenotype in 16cell cysts (data shared below, but detailed in a separate manuscript currently under review at *Developmental Biology*). This indicates that expression of the EcR^{F645A} and EcR^{DBL} alleles do yield phenotypes in the germline, but specifically fail to phenocopy the block to differentiation seen by expressing the wildtype EcR and EcR^{W650A} alleles.

Reviewer 1 says "*it is likely suggesting that there's no ecdysone signaling/activity in GSC and that EcR RNAi phenotype could be due to that EcR/USP functions as a transcriptional repressor in GSC without ecdysone binding*". We agree with this comment and, indeed, our model is that EcR/Usp does not require ecdysone binding to exert its repressive function in GSCs. While our gain-of-function experiments have the usual caveat that the protein is expressed at non-physiological levels, they are nonetheless useful for interrogating how germ cells respond to derepressed EcR signaling. Our findings support the model that that EcR acts as a repressor in GSC/CBs, where ecdysone levels are low and co-repressor levels are high, but becomes an activator in differentiating germ cells as the co-repressor levels decline. This fits with the field's current model of EcR activation (Morrow and Mirth, 2024; Kreher et al., 2017; Hu et al. 2003).

Our finding that the three "dominant negative" alleles (EcR^{W650A}, EcR^{F645A}, and EcR^{DBL}) do not fully phenocopy each other hints at an additional layer of complexity that we are currently investigating in a separate study. We postulate that the phenotypic differences we see in the germline likely indicate precise control over EcR repression/depression/activation during germ cell differentiation. We are keen to explore these mechanisms in-depth in our future work. We are currently designing new reagents to manipulate co-repressor expression and block co-regulator binding to EcR, to probe the EcR/co-repressor interaction, and to report EcR transcriptional activation and repression. As these experiments require building and testing new transgenic lines, we feel they are outside of the scope of this manuscript, which instead takes advantage of the EcR germline over-expression phenotype and its similarity to manipulation of BMP signaling to build a community resource for analysis of undifferentiated germ cells.

2. **Clarity and Accessibility of the Manuscript.** All reviewers note, to varying degrees, that:

- The manuscript would benefit from clearer explanations, especially of allele behavior and signaling logic.
- Figures, annotations, and terminology assume significant prior knowledge, limiting accessibility to a broader audience.
- Several conclusions are stated more strongly than the data currently justify.

We appreciate the Reviewers' suggestions to make the manuscript clearer for a broader audience, and to more carefully interpret our data (particularly in inferring causal relationships between EcR and BMP signaling, which are not well-justified by our data). We outline our changes to the text in the point-by-point response below, but to summarize, we've added more annotations to the figures, shortened/clarified the text, and included additional explanations of the signaling logic (including a revised model) throughout the Results and Discussion. We hope this makes the manuscript more accessible for a general audience.

DEVELOP-S-25-00994 – Point by Point Response. (Our responses are in blue text.) REVIEWER 1

Major concerns:

1. Line 147 describes the EcR expression in GSCs (Figure 1B). It is unclear whether the antibody staining in GSCs is real signal for EcR protein and needs validation. An easy experiment is to compare this to nos>EcRRNAi germarium to see whether EcR staining is indeed knocked down.

We appreciate and share the reviewer's concern. Indeed, in our hands, the EcR antibodies are inconsistent between experiments and antibody batches. Since previous studies used a different antibody and concluded that EcR was expressed in all cell types in the ovary (Buszczak et al 1999, Development 126: 4581–4589), we elected to remove the EcR antibody experiment from the

© 2026. Published by The Company of Biologists under the terms of the Creative Commons Attribution License (https://creativecommons.org/licenses/by/4.0/).

revised manuscript.

2. Line 152-153 described "EcRRNAi1, which targets all EcR isoforms; Figure S1A-C". This is confusing and inconsistent with Figure S1A showing that EcRRNAi1 targets EcR.A isoform. In addition, Figure S1C showed that EcR.B1 isoform level is also affected by EcRRNAi1. It is very confusing why EcRRNAi1 can affect EcR.B1 level if it targets EcR.A isoform specifically according to the hairpin structure. In addition, EcRRNAi3 showed no detectable EcR mRNA level in the entire ovaries, which is inconsistent with the fact that somatic follicle cells should have EcR mRNA expression. This leads the reviewer to question the validity of this method to validate the RNAi efficacy using the ovaries, since the cell composition in the ovaries are potentially different in different genotypes.

While we do not fully understand why, we suspect that self-regulation of EcR may be a contributing factor to a lack of isoform specificity in the RNAi lines. EcR can activate its own transcription (Karim & Thummel 1992; Romani et al 2015), thus we postulate that lack of EcR-A also depletes EcR-B in the germline. The reviewer is correct to point out that our description of the RT-PCR experiment used to validate the specificity of the EcR RNAi tools was not sufficient, and we included more details for this experiment in the Supplementary Information (Figure S1 legend). We performed this assay using a semi-quantitative PCR, assuming that 1) *EcR* mRNA is present in somatic follicle cells in the ovary, 2) the cellular composition and size of *nos>EcR RNAi* ovaries are roughly equivalent to wild type, and 3) nurse cells produce high levels of *EcR* mRNA. Ovary size and egg chamber composition were roughly equivalent between wild type and experimental samples, so we extracted mRNA from whole ovaries. Thus, wild type ovaries represent the level of *EcR* mRNA that is equivalent to somatic mRNA + germ cell mRNA. When we visualized *EcR* mRNA at higher cycle numbers (32-35 cycles), *EcR* mRNA could be detected in all of the *nos>EcR RNAi* experimental samples, which we assumed was from follicle cells as the reviewer indicated. At lower PCR cycle number (the gel image presented is at 24 PCR cycles), *EcR* mRNA was readily detected in the wild type sample, but greatly reduced in the *nos>EcR RNAi* experimental samples, confirming that less *EcR* mRNA is made in the germline in the presence of the RNAi. We were surprised to see that EcR^{RNAi1}, which we designed to target the *EcR-A* isoform, depletes *EcR* mRNA in a non-specific manner. Because of this, we were careful to avoid any descriptions suggesting isoform specificity in the RNAi lines. The fact that all three *EcR RNAi* lines produce similar phenotypes also argue against any isoform specificity. For example, females from all three *EcR RNAi* lines produce less eggs than wild type flies (new Supplemental Figure S1D).

3. Figure 1 showed EcRRNAi1 affects the GSC number; however, it is confusing why FLP clone system (Figure 1J) is stronger than the constitutive Gal4 knockdown (Figure 1G). In addition, EcRRNAi2 and EcRRNAi3 seemed more efficient than EcRRNAi1 (Figure S1), it is necessary to use these RNAi lines to recapitulate the phenotype, particularly considering that EcRDN does not show the GSC loss phenotype.

We appreciate the reviewer's comments about the additional *EcR RNAi* lines. We performed similar analyses as displayed in Figure 1G using the other two EcR RNAi lines, but did not include them in the main figure to improve clarity in the figure. We have now added the additional GSC timecourse analysis on all three *EcR RNAi* lines (new Supplemental Figure S1F), as well as a static timepoint comparing GSC number per germarium across the *EcR RNAi* lines (new Supplemental Figure S1E). Although the phenotype is mild, it is consistent between independent RNAi lines and statistically significant in each case. We think that the apparent differences in the strength of the phenotype between the timecourse assay and the FLP-inducible clonal assay is due to the age of the flies. In the timecourse, we collected females at eclosion and aged them in parallel to wildtype over 10 days (new Figure 1D). In the FLP-inducible assay, females were collected 2-3 days after eclosion, heat shocked for 3 days, and monitored over the course of 14 days (new Figure 1G). Flies in the mosaic assay labeled as "14DAHS" are actually more than a week older than the latest timepoint in the timecourse assay. It is also possible that the clones in the FLP-inducible assay are lost faster because they are actively being outcompeted by neighboring wildtype GSCs.

© 2026. Published by The Company of Biologists under the terms of the Creative Commons Attribution License (https://creativecommons.org/licenses/by/4.0/).

4. There's no explanation why EcRDN lines do not show GSC loss in Line 160-162. If results are indeed repeatable, it is likely suggesting that there's no ecdysone signaling/activity in GSC and that EcRRNAi phenotype could be due to that EcR/USP functions as a transcriptional repressor in GSC without ecdysone binding. A way to differentiate these scenario is to use ecdysone ligand sensor to determine whether ecdysone ligands can be detected in GSCs.

We agree with the reviewer that the difference in GSC loss phenotypes between the EcR RNAi lines and the EcR^{F645A} and EcR^{DBL} transgenes likely indicates that EcR functions as a transcriptional repressor in GSCs, and that depletion of receptor titer, rather than activation function, explain why the EcR^{F645A} and EcR^{DBL} transgenes do not cause GSC loss. We include additional conclusions in the revised text to emphasize this point (pg. 5, line 147-148). We attempted to use an ecdysone ligand sensor to test the availability of ligand in GSCs; however, none of the ligand sensors or transcriptional reporters for EcR were built in transgenic vectors that permit stable reporter expression in mitotically-dividing germ cells. (Most UAS-based vectors included promoter elements from *hsp70*, which is degraded by piRNAs in early germ cells, and 3'UTRs from either *nos* or the viral SV40 gene, which are actively turned over in early germ cells.) We are currently building new reporters and new Gal4 drivers for early germ cells, but these will require extensive testing before they can be used in this context and are thus not feasible for us to include in this manuscript.

We also note here that expression of the EcR^{F645A} and EcR^{DBL} alleles do yield phenotypes in the germline, but specifically fail to phenocopy the block to differentiation seen by expressing the wildtype EcR and EcR^{W650A} alleles. In a separate study (currently under review at *Developmental Biology*), we demonstrate that the EcR^{F645A}, EcR^{DBL}, and EcR RNAi transgenes share an encapsulation delay phenotype in 16cell cysts (data shared at right from the manuscript currently under review; this phenotype can also be seen in new Figure 1C of the current manuscript). This likely indicates precise regulation over EcR repression/ derepression during germ cell differentiation, and we are exploring this intriguing mechanism in future work.

NOTE: Figure provided for reviewer has been removed. It showed Figure 1 from SWAIN, LINDSAY A., PHIPPS, DANIEL N., WEBSTER, SUSANNAH CATHERINE G., JUNG, LAUREN E. and ABLES, ELIZABETH T. (2026) Germline cysts require Ecdysone Receptor for proper timing of encapsulation in the Drosophila ovary. DEVELOPMENTAL BIOLOGY. doi: https://doi.org/10.1016/j.ydbio.2026.06.008. We have removed unpublished data that had been provided for the referees in confidence.

5. The conclusion that "Over-expression of EcR in GSCs and cystoblasts delays germ cell differentiation in a ligand-dependent manner" in Line 168-169 is not supported by the experimental data. The experimental data is rather confusion and conflicting in Figure 2.
   I. Authors showed that EcR-B1W650A blocked germline differentiation, while EcR-B1F645A and EcR-B1DBL did not in Line196-199. Authors postulate that EcR-B1F645A/EcR-AF617A functions as ligand sponges to affect endogenous ecdysone level. If that is the case, EcR- B1DBL cannot bind ligand and should show the same phenotype as EcR-B1W650A, which is completely opposite (Figure 1J).
   II. In addition, authors used the ecdysone feeding to partially rescue EcR-B1 phenotype to support their claim; however, it will be important to show that ecdysone feeding cannot rescue EcR-B1W650A phenotype as EcR-B1W650A cannot bind to ecdysone.

We thank the reviewer for pointing out that ligand binding alone cannot fully explain the different phenotypes we see between EcR^{F645A}, EcR^{W650A}, and EcR^{DBL}. In fact, the original analysis of these mutations to the ligand binding domain by Cherbas and colleagues (Hu et al Cherbas, 2003) concluded that the EcR^{F645A} allele cannot switch from a repressed to a derepressed/active transcription due to improper interactions with co-regulators. We speculate that it is this interaction, and not ligand binding per se, that more likely explains the phenotypic differences we see (pg. 6, lines 181-189). Further, we agree that the ecdysone feeding experiment is insufficient

© 2026. Published by The Company of Biologists under the terms of the Creative Commons Attribution License (https://creativecommons.org/licenses/by/4.0/).

to help explain the phenotypic differences between EcR^{F645A}, EcR^{W650A}, and EcR^{DBL} and is not well-controlled. We elected to remove this confusing data from the manuscript.

6. There's no detailed description of the re-clustering of undifferentiated germ cells. It is unclear the accuracy of the subsequent analysis. It seems that each clusters in different genetic backgrounds are very different. It will probably better to combine all the undifferentiated germ cells from all genotypes for re-clustering to find similar cells.

We apologize for the oversight in our description of the reclustering. We did combine all the undifferentiated germ cells across genotypes as the Reviewer suggests. We clarified this point in the text (pg. 8, lines 224-226).

7. Line 259-260 described that tkv "was up-regulated in response to both EcR-B1 and tkvAct over-expression (Figure 4H)". It is not so obvious that tkv was upregulated in both EcR-B1 and tkvAct over-expression in comparison to bamΔ86 mutant. In the subsequent Tkv antibody analysis (Figure 4K-M), it will be better to include bam Δ86 mutant to confirm this conclusion and support that EcR-B1 over-expression indeed upregulates Tkv expression.

The Reviewer's point is well-taken. We repeated this experiment with the *bam* RNAi as an additional control and included this data (new Figure 4N).

8. In line 430-432, it is unclear why authors tested Lola-DE isoform. In addition, the data are conflicting to each other. Why did anti-Lola-DE staining show different expression pattern as lola-DE reporter line? How can these data support the conclusion that Lola influence gene expression programs during germ cell differentiation.

In our final analysis, we used the undifferentiated cell transcriptomics data to ask whether there were any notable transcriptional programs at work in the earliest stages of germ cell differentiation. We used the SCENIC algorithm to predict transcriptional networks guided by a primary transcription factor that would explain the groups of genes up- and down-regulated in each cluster (Figure 7A). Although most of the transcription factors that appeared in the dataset are completely uncharacterized in the germline, Lola, Dp, Ovo, and Nej stood out for their prior characterization in female or male germ cells. Since *ovo* and *nej* expression patterns were published elsewhere, we sought to include the endogenous expression patterns for *dp* and *lola*. While the expression data for *dp* is consistent with its role as a cell cycle regulator, the expression data for *lola* is, as the Reviewer points out, very confusing. We tested the reagents for *lola* that were available; namely, an antibody from the Developmental Studies Hybridoma Bank (reported to be targeted against isoform DE) and several Lola-GFP transcriptional reporters.
Given the complexity of the *lola* locus (ie, >20 mRNA/protein isoforms), more detailed loss-of-function analyses for Lola will be necessary to make any conclusions about its role in the germline. We attempted to leave this as a "future directions" hypothesis; we've now toned down our conclusions and speculate about the activity of Lola in the germline (pg. 14, lines 409-412).

Minor concerns:

1. Line 147-149 concludes that EcR levels were lower in undifferentiated germ cells than in 16-cell cysts and lower in the germline compared to somatic follicle cells. It is not obvious from the images shown in Figure 1B-C. Quantification is required to substantiate this statement.

We removed this data. See major point #1 above.

2. It will be better to replace EcRRNAi to EcRRNAi1 in Figure 1F-G and Figure 1I-J to increase the clarity.

Thanks for catching this. We fixed the labels in new Figure 1C-D,F-G.

3. Line 231-232 indicates HP6 and minidiscs to differentiate undifferentiated and differentiated

© 2026. Published by The Company of Biologists under the terms of the Creative Commons Attribution License (https://creativecommons.org/licenses/by/4.0/).

germlines, respectively (Figure S3). Figure S3 did not show minidiscs expression. Please add the expression panel to confirm the clustering.

This data is now in new Supplemental Figure S4F. We also included the scRNAseq data for Blanks and Chinmo (Figure S4D-E), to accompany the antibody data in Figure 3.

4. In Figure 5, the cluster-enriched genes are not obviously show in specific clusters. An outline of each cluster may help for better visualization.

Thanks for the suggestion. We added color-coded lines around each cluster in Figure 5 to help improve the visualization.

5. There's no supplemental dataset to support the analysis in Figure 6A, as well as Figure 6H and 7A.

This was an oversight on our part. We've now uploaded three Supplementary Datasets (Table S2-S4) to provide the original markers analysis, the undifferentiated germ cells marker analysis, and the SCENIC data to which the reviewer refers.

REVIEWER 2

"Moderate" concerns:

1. Lines 147-149 and Figure 1B,C: In general, different EcR expression levels are evident in the representative images, but this could be supported better by quantification. Additionally, there is a discrepancy when comparing somatic versus germline EcR. The text states "...lower in the germline compared to somatic follicle cells until stage 10...", but the staining in the germline seems to be higher than in follicle cells in the region 3 and stage 1 egg chamber shown in Fig 1.

This was Reviewer 1's concern as well, and we refer the Reviewer to their point #1 above. We removed this data.

2. Lines 299-322 and Figure 5: Assessing expression patterns of the genes shown in the mutant backgrounds would further confirm the transcriptomics data. I do understand that doing so for all reporter-background combinations might be unreasonable, but a subset might be feasible.

We appreciate the Reviewer's point, and we tried to assess the expression pattern in the three "tumor" backgrounds as much as possible. The proteins used to compare genotypes in Figure 3 and 4 were selected in part because they were differentially expressed in the scRNAseq datasets. (See also new Supplementary Figure S8, where we tested *mov10* and *Rbfox1*. We moved the text explaining this data earlier, per the Reviewer's minor concern #3 below.) Validating additional genes from the scRNAseq data in the EcR and Tkv over-expression alleles turned out to be more challenging than we anticipated because of two issues. First, as you will see in Figure 5 and S8, *mov10* is endogenously expressed in GSC/CB/2c, and is expanded in the EcR over-expression background. But, is this because the cells are blocked from differentiating, or because *mov10* is up-regulated? It is not possible to distinguish between these possibilities. Second, we tried using the large catalog of CRIMIC Gal4-based expression lines available in the stock center to determine the expression of uncharacterized genes from the scRNAseq datasets. We discovered, however, that while the CRIMIC tools are extremely useful for somatic cells, they do not produce stable Gal4 expression in GSC/CB/2c cells (this is a perpetual problem in the field).

Minor concerns

1. Figure 1F: The anterior-most tip of the germarium shown appears to be tucked under, thus not including the niche.

© 2026. Published by The Company of Biologists under the terms of the Creative Commons Attribution License (https://creativecommons.org/licenses/by/4.0/).

Keeping in mind a comment from Reviewer 3, asking for additional annotations to make the image accessible for a broader audience, we've added asterisks to the image (new Figure 1B-C, E-F) to indicate the position of the niche. (In panel 1C, the niche is slightly off to the side of the germarium.)

2. Figure 2: The order of manipulations shown for the representative images is not the same as the order shown for the quantification.

We re-arranged the graph (new Figure 2J) per the Reviewer's suggestion.

3. Related to "Moderate concern" 2: The data describe in lines 339-344 and shown in Figure S7 addresses the moderate concern 2, and may fit better with that section.

We agree and modified the text per the Reviewer's comment. This section is now on pg. 10, lines 305-313.

4. Figure 7F: The colors referred to in the figure legend do not match with the actual model schematic. Additionally, there is some disconnect between what is described and the "signal bars" in the stem versus egg cell. Specifically, the bars in the stem cell should be filled in for the "stem cell" genes and empty for the "egg" and the reverse should be true for the egg cell. Instead, this is what is shown for the egg cell.

Thanks for pointing this out. Keeping with the requests by Reviewers 1 and 3 to more clearly explain the model, we redesigned the schematic and amended the legend.

REVIEWER 3

Major concerns

Interpretation of EcR allele results. I found it difficult to fully interpret the different results that were observed with the different EcR alleles in both GSC maintenance and germ cell differentiation. In some cases, it would be beneficial for the authors to explain their interpretation of the differences they observe. In other cases, I found the explanations confusing and could either use clarification or reinterpretation. For instance:

1. For the GSC maintenance experiments in Figure 1, how do the authors interpret the differences between the EcR RNAi effect on GSC number vs. the failure of either the activation mutants (F645A) or double mutants with the ligand binding mutant (DBL) to produce a similar phenotype? It's unclear to me how to interpret these differences. Was the W650A allele examined on its own in this assay?

The point is well-taken, and we refer the Reviewer to our response to Reviewer #1 (point 4) above. We added a more thorough interpretation to the text. We did not examine the EcR^{W650A} allele in this assay because like the over-expression of wildtype EcR, the EcR^{W650A} produces so many single-cell germ cells that it is very difficult to assess GSC number specifically.

2. I found the description of the experiments to examine EcR allele effect on germ cell differentiation and subsequent interpretation in lines 193-203 to be confusing. I believe that because OX of EcR-B1 along with the binding defective allele (W650A) both limit differentiation, whereas F645A (activation defective) and DBL (both binding and activation defective) don't limit differentiation led the authors to: "…postulate that they [unclear what "they" refers to… EcR-B1OX and W650A?] function as ligand sponges, effectively shunting endogenous EcR activity towards transcriptional repression." But it's unclear to me how the

© 2026. Published by The Company of Biologists under the terms of the Creative Commons Attribution License (https://creativecommons.org/licenses/by/4.0/).

binding defective allele acts as a ligand sponge and why the F645 allele, which still retains binding, and so could act as a ligand sponge, doesn't produce a phenotype. Is the phenotype resulting from EcR-B1 over-expression due to increased expression of endogenous EcR target genes? Or increased suppression of endogenous EcR target genes through the additional EcR- B1 sequestering ligand from endogenous sites? While rescue of EcR-B1 expressing larvae with endogenous ecdysone might support the sponge hypothesis, would you expect ecdysone feeding to rescue the W650A allele, which can't bind ecdysone? It seems like there are some inconsistencies that belie a simple explanation here and it would be helpful to have a clearer sense of how we can interpret the EcR alleles, especially EcR-B1 over-expression.

We agree with the Reviewer that the distinction between the EcR^{F645A} and EcR^{W650A} alleles is important, and we did not adequately address (or appreciate) the nuances between the alleles in the original manuscript. As described in the review summary above, we carefully considered the original descriptions of the EcR^{F645A} and EcR^{W650A} alleles from the Cherbas lab (Hu et al., 2003). Based on their biochemical data, Cherbas and colleagues indicated that the EcR^{F645A} and EcR^{W650A} alleles also likely differ in their ability to interact with co-regulators. Because we show that the co-repressor Smrter is enriched in GSC/CB/2c germ cells, we postulate that EcR binds Smrter in these cells, then is derepressed when ligand concentration rises and Smrter concentration falls, and the complex switches to a transcriptional activator. In the context of our data, this would explain why a loss of total EcR yields a phenotype in GSCs, but over-expression of the EcR^{F645A} and EcR^{DBL} alleles (which phenocopy each other in every assay we have tested) simply reinforces EcR target repression in the low-ligand environment. In contrast, over-expression of the wildtype and the EcR^{W650A} alleles may create an excess of unliganded receptor that cannot transactivate target genes, or may derepress the receptor from target genes prematurely. Interestingly, this blocks germ cell differentiation. In a perfect world, we would use a cystoblast-specific Gal4 driver to test the distinction between GSC maintenance and germ cell differentiation, and we attempted to do this by using the bam-Gal4 driver (new Supplementary Figure S3A-C,F). However, we were surprised to see that over-expression of the wildtype EcR under bam-Gal4 was not sufficient to block differentiation. We believe this is due to the timing of driver activation, as bam-Gal4>mCherry-EcR could not be detected until 4/8c cysts (new Supplementary Figure S3B). We've attempted to better reconcile these interpretations in the revised text (abstract; intro pg. 4, lines 120-129; results pg. 6, lines 174-189; discussion pg. 14, lines 420-428) and in a new model (Figure 7F).

3. This issue carries over into the subsequent expression analysis to identify "ecdysone responsive genes" through the comparison of EcR-B1OX vs tkv[ACT], and bam[delta86]. especially in thinking about whether the gene expression changes observed reflect a direct effect of EcR activation of the target vs. indirect activity of EcR-B1 OX leading to endogenous EcR producing transcriptional repression. It also comes up in the summary Figure 7F… does EcR/USP activate Tkv? Or is the increase of Tkv in the EcR-B1 Ox an indirect effect of increased EcR repression at endogenous sites? A better clarification and explanation of how these alleles are acting would make these results easier to interpret and clarify the model for a general audience.

We appreciate the Reviewer's comments and assume they are referring to the Results section beginning line 349 and Figure 6 (as well as the model in Figure 7F). The Reviewer is correct in their assessment that we cannot delineate genes that are transcriptionally repressed versus activated by EcR from our scRNAseq dataset. We recognize that a limitation of the over-expression is that some genes may be inappropriately temporally activated in this model. However, EcR over-expression, Tkv over-expression, and bam mutants all enrich ovaries for large numbers of undifferentiated germ cells. These are cell types that are typically very low abundance in the tissue and have been largely over-looked by prior scRNAseq atlases. While other studies have used bulk RNAseq approaches to catalog gene expression in undifferentiated germ cells (ie the bam mutants or the Tkv over-expression), our study combining these genetic backgrounds with the EcR over-expression background gives us a unique opportunity to search for fine resolution differences in gene expression between differentiation states. By examining ovaries enriched for GSC/CB/2c-like cells, we now see expression of canonical ecdysone- responsive genes

© 2026. Published by The Company of Biologists under the terms of the Creative Commons Attribution License (https://creativecommons.org/licenses/by/4.0/).

in the early germline, suggesting an endogenous ecdysone response. Moreover, we included the analysis of potential transcriptional regulators of each of the undifferentiated germ cell clusters in the *bam* mutants alone specifically because we did not modify EcR in this genetic background. The fact that ecdysone-responsive transcription regulators (Ftz-f1, EcR, Smrter, Eip78C, Hr38) are computationally defined as potential modifiers of gene expression in the undifferentiated (*bam* mutant) cells supports our argument that there is an endogenous ecdysone response in these germ cells (Figure 6H). Taking the Reviewer’s comment to heart, we revised the text in several places to substitute the word “activation” for “regulation” to better explain what we can and cannot infer from this data with regards to the mechanism of EcR binding/activation, and revised the model accordingly.

4. While EcR-B1 over-expression produces a qualitatively bam phenotype independently of tkv or dpp, is the converse true? Does the bam or tkv[ACT] phenotype depend on EcR signaling? Is it suppressed by EcR DN or RNAi? Does the result described in lines 382-384, Figure 6H suggest that the bam mutant phenotype might depend on EcR activity?

This is a great question. We attempted to test this in by modulating *dpp* levels or EcR signaling in the opposite genetic background (see new Supplemental Figure S5), and by this albeit crude genetic analysis, we do not think that the *tkv* or *bam* phenotypes depend on EcR. We believe there are some limitations to this analysis, however, due to the strength of the germline Gal4 driver that we used. Unfortunately, it is really the only driver we have for these cells at this time, so we did not pursue this question at this time due to the technical limitation.

Minor concerns

1. Line 152 says that EcR RNAi1 targets all EcR isoforms. However, in figure S1 it appears that the EcR RNAi1 would be targeted to the EcR A isoform and the RNAi2 and RNAi3 target the common exons. Am I misinterpreting this figure?

No, the Reviewer is not misinterpreting the figure. We are not exactly sure why, but we see depletion of both EcR-A and -B isoforms in the EcR RNAi1, even though it was designed to target EcR-A. We speculate that this is due to the regulation of EcR by itself. Please see point #2 in response to the same comment by Reviewer 1 above. We added our interpretation of the RNAi isoform specificity to new Supplemental Figure S1 legend.

2. For clarity, I think the mCherry-tagged EcR-A and B1 alleles in Figure 2 could be moved into supplemental data or omitted, I don't think the analysis of those alleles is necessary for the overall conclusions.

We appreciate the Reviewer’s suggestion, but respectfully disagree. This data reinforces our conclusions because we see very similar germline phenotypes with independent transgenic manipulations. This is the reason we also included the EcR-A alleles in the study, even though we did not use these for scRNAseq.

3. Line 114, suggest "received by GSCs via".

Thanks for catching this. The new phrasing is now pg. 4, lines 110-111.

4. Line 149, should "until" be "at"? after stage 10 is germline expression equal/higher than follicle cells?

We removed this data, considering the comments by Reviewer 1 (point 1) and Reviewer 2 (point 1) above.

© 2026. Published by The Company of Biologists under the terms of the Creative Commons Attribution License (https://creativecommons.org/licenses/by/4.0/).

5. For accessibility, it would be helpful to label the components of the stage 10 image in Figure 1C for a general audience.

Thanks for the suggestion. We have included more labels throughout the figures, to make them more accessible for a broad audience.

6. In Figure 1 E-H it would be good to label cells with arrows/arrowheads since the green and purple label multiple cell types.

Great suggestion. We actually expanded this to all of the immunofluorescence images, such that cap cells (asterisks), GSCs (arrowheads), and differentiated germ cells (arrows) are all annotated.

7. In Figure S4, it would be helpful to a general audience if the authors could better annotate the phenotype that is being evaluated in these images.

We appreciate the reviewer's comment. We couldn't identify a great way to annotate this phenotype, since the important part is that all of these manipulations make tiny ovaries with no mature egg chambers. We expanded the figure legend (new Figure S5) to address the reviewer's concern.

Second decision letter

MS ID#: dev.205460R1

MS TITLE: Ecdysone Receptor autonomously controls germ cell differentiation in the Drosophila ovary

AUTHORS: Lauren E. Jung; Alexandria I. Warren; Changhong Yin; Weihua Huang; Allison C. Simmons; Samantha McDonald; Lindsay A. Swain; Victoria E. Garrido; Daniel N. Phipps; BiClaireline Cesar; Danielle S. Finger; Zhipeng Sun; Todd G. Nystul; Elizabeth T. Ables

Dear Dr Ables,

I am happy to tell you that your manuscript has been accepted for publication in Development, pending our standard publication integrity checks.

Reviewer 2

I appreciate that the authors put in the time and effort to thoughtfully and thoroughly address my concerns about the original submission. The have satisfactorily addressed my comments.

Reviewer 3

SUMMARY OF THE ADVANCE MADE IN THIS PAPER AND ITS POTENTIAL SIGNIFICANCE TO THE FIELD

The work in this manuscript describes how hormonal signals can mediate distinct responses in different cell types within a tissue to coordinately regulate tissue function (in this case, GSC maintenance and differentiation in the Drosophila ovary). This work provides insight into a valuable experimental model that could provide paradigms for understanding hormonal regulation of tissue function in many different systems.

SUGGESTIONS TO AUTHORS

© 2026. Published by The Company of Biologists under the terms of the Creative Commons Attribution License (https://creativecommons.org/licenses/by/4.0/).

I appreciate the responses that authors have made to my suggestions on the original manuscript and I believe these changes have addressed my primary concerns by: 1. creating more transparency about their interpretation the different EcR alleles with respect to their known molecular functions, and 2. Providing additional explanations and information in the figures to orient a broader range of readers, which should increase the impact of these findings. I recommend that this revised manuscript is now acceptable for publication and look forward to it being shared with the broad readership of Development.

© 2026. Published by The Company of Biologists under the terms of the Creative Commons Attribution License (https://creativecommons.org/licenses/by/4.0/).
